# Senolytics prevent mt-DNA-induced inflammation and promote the survival of aged organs following transplantation

**DOI:** 10.1038/s41467-020-18039-x

**Published:** 2020-08-27

**Authors:** Jasper Iske, Midas Seyda, Timm Heinbokel, Ryoichi Maenosono, Koichiro Minami, Yeqi Nian, Markus Quante, Christine S. Falk, Haruhito Azuma, Friederike Martin, João F. Passos, Claus U. Niemann, Tamara Tchkonia, James L. Kirkland, Abdallah Elkhal, Stefan G. Tullius

**Affiliations:** 1 Division of Transplant Surgery, Department of Surgery, Brigham and Women’s Hospital, Harvard Medical School, Boston, Massachusetts USA; 2 Institute of Transplant Immunology, Hannover Medical School, Hannover, Lower Saxony Germany; 3 Department of Nephrology, Charité Berlin, Berlin, Germany; 4 Department of Urology, Osaka Medical College, Osaka, Japan; 5 Department of Urology, The Second Xiangya Hospital, Central South University, Changsha, China; 6 Department of General, Visceral and Transplant Surgery, University Hospital Tübingen, Tübingen, Germany; 7 Department of General, Visceral and Transplant Surgery, Charité Berlin, Berlin, Germany; 8 Robert and Arlene Kogod Center on Aging, Mayo Clinic, Rochester, MN USA; 9 Department of Physiology and Biochemical Engineering, Mayo Clinic, Rochester, MN USA; 10 Department of Anesthesia and Perioperative Care, University of California San Francisco, San Francisco, CA USA; 11 Division of Transplantation Department of Surgery, University of California San Francisco, San Francisco, CA USA

**Keywords:** Inflammation, Dendritic cells, Transplant immunology, Ageing

## Abstract

Older organs represent an untapped potential to close the gap between demand and supply in organ transplantation but are associated with age-specific responses to injury and increased immunogenicity, thereby aggravating transplant outcomes. Here we show that cell-free mitochondrial DNA (cf-mt-DNA) released by senescent cells accumulates with aging and augments immunogenicity. Ischemia reperfusion injury induces a systemic increase of cf-mt-DNA that promotes dendritic cell-mediated, age-specific inflammatory responses. Comparable events are observed clinically, with the levels of cf-mt-DNA elevated in older deceased organ donors, and with the isolated cf-mt-DNA capable of activating human dendritic cells. In experimental models, treatment of old donor animals with senolytics clear senescent cells and diminish cf-mt-DNA release, thereby dampening age-specific immune responses and prolonging the survival of old cardiac allografts comparable to young donor organs. Collectively, we identify accumulating cf-mt-DNA as a key factor in inflamm-aging and present senolytics as a potential approach to improve transplant outcomes and availability.

## Abstract (web summary)

Organ transplantation involving aged donors is often confounded by reduced post-transplantation organ survival. By studying both human organs and mouse transplantation models, here the authors show that pretreating the donors with senolytics to reduce mitochondria DNA and pro-inflammatory dendritic cells may help promote survival of aged organs.

## Introduction

The world population is aging rapidly, with the population that is over 60 years growing faster than any younger age segment. Indeed, those over 60 years will surpass one billion by 2020^1^. Organ transplantation is the treatment of choice for patients with irreversible end-stage organ failure. The supply of organs, however, is limited, resulting in prolonged waiting times with many patients dying or becoming too ill to be eligible for transplantation^2^. Currently, the most obvious strategy with potential for closing the gap between demand and supply would be to enable the use of organs from older deceased donors that currently are frequently discarded^3^. We have shown in preclinical and clinical studies that increased donor age poses a significant risk for adverse outcomes, such as more frequent acute rejections of renal allografts^4,5^. Moreover, recovery after IRI is compromised in older organs, clinically translating into higher rates of delayed graft function^2,6^.

Aging is associated with increased senescent cell burden^7^ that is linked to chronic, low grade, sterile inflammation^8–10^. Of interest, we recently reported that transplanting even small numbers of senescent cells into young recipients causes functional impairment^11^. Increased levels of cytokines, including IL-6, IFN-γ, and TNF-α, contribute to the pro-inflammatory secretome of senescent cells, termed the senescence-associated secretory phenotype or SASP^12^. Accumulation of senescent cells with aging has been shown to contribute to declining renal function and cardiac stress resilience, in addition to cardiac hypertrophy/fibrosis, a process that is further exacerbated by mitochondrial dysfunction^11–15^. Damage-associated molecular patterns (DAMPs), which include mt-DNA, also increase with aging^16^. Relationships among senescent cell accumulation, mt-DNA, the SASP, outcomes of transplantation in clinically relevant disease models, and the potential to mitigate injury and augmented immunogenicity of older organs by targeting senescent cells have so far not been tested^17–21^. However, recent studies propose that circulating mitochondria and mt-DNA might mediate early allograft dysfunction^22,23^.

IRI is characterized by initial tissue hypoxia with metabolic changes and subsequent further damage with the reintroduction of oxygen and elevated shear forces during reperfusion. Local tissue injury is followed by a systemic sterile inflammatory response, mediated by DAMPs including mt-DNA. DAMPS, in turn, stimulate immune responses through pattern-recognition receptors, including endosomal Toll-like receptor 9 (TLR9)^24^. IRI is of clinical relevance in numerous illnesses, particularly in the elderly^25–27^. Delineating underlying changes in tissue homeostasis during stress-surveillance responses in aging is thus of critical clinical importance and highly relevant to multiple diseases and conditions, including transplantation.

We have previously identified old DCs as the main source of augmented alloimmune responses and accelerated rejection of old cardiac allografts via Th17-driven alloimmune responses^5^. Indeed, old DCs exhibit a pro-inflammatory profile, with secretion of pro-inflammatory cytokines (i.e., IL-6, IFN-γ, and TNF-α)^28^.

Here, we demonstrate in clinical and experimental models that DC activation in aging is caused by increased systemic levels of cell-free mt-DNA communicated through TRL9, which promotes Th1/Th17 immunogenicity following ischemia reperfusion injury. Moreover, we identify senescent cells as a key source of cf-mt-DNA. Finally, we show that treatment with the senolytics, Dasatinib plus Quercetin (D + Q), which selectively eliminate senescent cells^29^, reduces senescent cell burden, cf-mt-DNA levels, alleviates age-associated inflammation, and specifically prolongs survival of cardiac allografts from old mice.

## Results

### DCs from old exhibit increased markers of activation

**Fig. 1:**
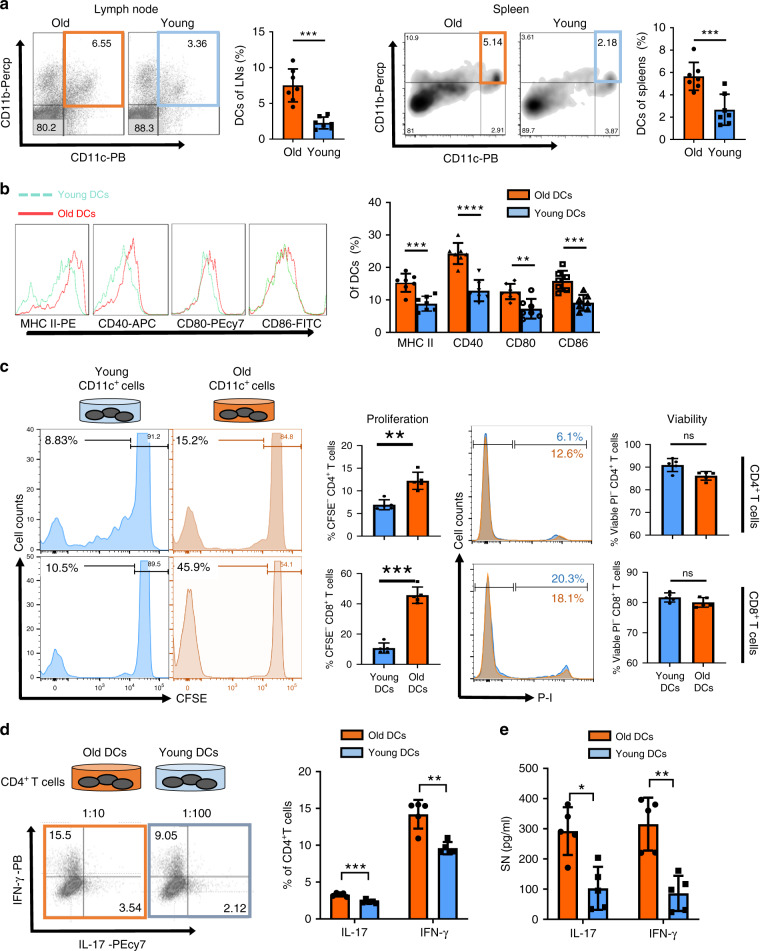
Dendritic cells from old mice exhibit an activated phenotype and promote Th1 and Th17 T cell responses. Single cell suspensions of lymph nodes and spleens from old and young C57BL/6 mice were labeled with anti-CD11c, anti-CD11b, anti-MHC class II, anti-CD40, anti-CD80, and anti-CD86. **a** The frequency of CD11b^+^CD11c^+^ DCs in lymph nodes (*p* = 0.0006) and spleens (*p* = 0.0023), and **b** the expression of costimulatory molecules (MHC-II: *p* = 0.0005/CD40: *p* < 0.0001/CD80: *p* = 0.0035/CD86: *p* = 0.0007) by splenic DCs were assessed by flow cytometry. **c** Proliferative capacities of CD4^+^ (*p* = 0.0079) and CD8^+^ T cells (*p* = 0.0079) co-cultured with DCs from old and young mice were determined using CFSE dilution and viability assessed using propidium iodide (**d**) CD4^+^ T cells from young mice were co-cultured with CD11b^+^CD11c^+^ DCs isolated from young and old mice and (**e**) pro-inflammatory cytokine expression was assessed by flow cytometry (IL-17: *p* = 0.0002/IFN-γ: *p* = 0.0013) and ELISA (Il-17: *p* = 0.004/IFN-γ: *p* = 0.0079); (*n* = 7 biologically independent animals), results are representative of at least three independent experiments. Column plots display mean with standard deviation. Statistical significance was determined by using two-tailed Mann–Whitney-test. Asterisks indicate *p* values *p ≤ 0.05, **p ≤ 0.01, and ***p ≤ 0.001, only significant values are shown. Source data are provided as a Source Data file.

We previously characterized the impact of aging on DC activation and CD4^+^ T cell fate and have shown that aging is associated with activated DCs and subsequent Th17-driven alloimmune responses^5^. Here, we found increased frequencies of peripheral lymphoid and splenic CD11b^+^CD11c^+^ DCs in old mice, findings that are in line with previous reports showing increased frequencies of circulating and lymphoid DCs (Fig. 1a). Next, we analyzed the impact of old mouse DCs on T cell responses and further delineated the phenotypic maturity of freshly isolated CD11b^+^CD11c^+^ DCs from young vs. old mice. Our results show elevated expression of MHC class II, in addition to enhanced expression of the costimulatory molecules, CD40, CD80, and CD86, in old but not young mouse DCs (Fig. 1b).

### DCs from old mice promote Th1 and Th17 immune responses

To delineate alloimmune responses, isolated CD11b^+^CD11c^+^ DCs from old and young C57BL/6 mice were co-cultured in a mixed leukocyte reaction with naive allogeneic (DBA/2J) splenic T cells to assess CD4^+^ and CD8^+^ T cell proliferation. Results of our carboxyfluorescein succinimidyl ester (CFSE) assay showed that CD11b^+^CD11c^+^ DCs from old mice significantly promoted accelerated proliferation of both CD4^+^ and CD8^+^ responder T cells compared with the stimulatory capacity of DCs from young mice. Of note, T cell viability assessed by propidium iodide (PI) was not significantly affected (Fig. 1c).

Collectively, these observations underscore augmented alloimmunity due to DCs from old mice.

With CD11b^+^CD11c^+^ DCs recognized as instigators of Th1- and Th17-driven inflammatory responses^30^, we next examined whether DCs from old mice co-cultured with DBA/2J CD4^+^T cells had a direct effect on Th1 and Th17 cell differentiation. Indeed, DBA/2J CD4^+^ T cells cultured with old mouse DCs had significantly higher levels (twofold increase) of IFN-γ and IL-17 production (Fig. 1d), suggesting age-specific Th1 and Th17 cell differentiation promoted by old mouse DCs. Consistent with these findings, supernatants of T cells co-cultured with old mouse DCs promoted enhanced production of IFN-γ and IL-17, as quantified by ELISA (Fig. 1e). These data demonstrate an age-specific activation of DCs that promotes Th1 and Th17 responses, two prominent CD4^+^ T cell pro-inflammatory subsets that are involved in IRI and transplant rejection^31,32^.

### Adoptive transfer of old DCs reduces allograft survival

**Fig. 2:**
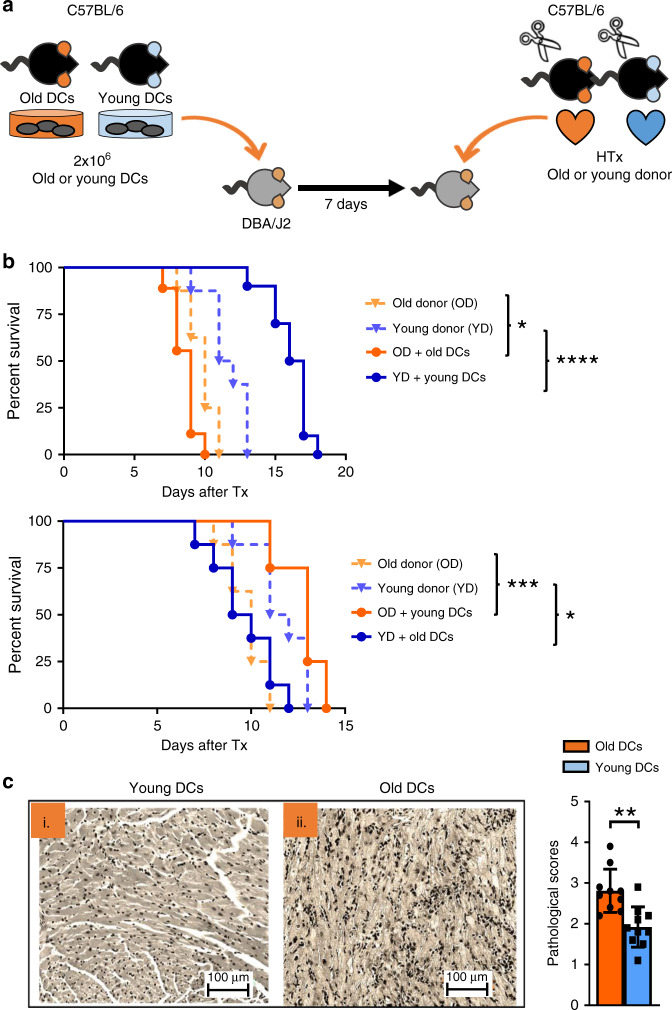
Dendritic cells from old mice impair cardiac allograft survival in young DBA/2J mice. **a** 2 × 10^6^ CD11b^+^CD11c^+^ DCs were sorted from old and young C57BL/6 mice and administered i.v. into young DBA/2J mice 7 days prior to allogeneic cardiac transplants. **b** Kaplan–Meier analysis of cardiac allografts from old and young C57BL/6 mice transplanted into untreated DBA/2J recipients compared with those that had received adoptively transferred CD11b^+^CD11c^+^ DCs from old and young B6 mice (old donor vs. old donor + old DCs (*p* = 0.0216)/+ young DCs (*p* = 0.0005) and young donor vs. young donor + young DCs (*p* < 0.0001)/+ old DCs (0.0182); (*n* = 8 biologically independent animals). **c** By day 11 after transplantation, grafts were procured, perfused, and 5 mm sections were stained with H&E for pathological evaluation (*p* = 0.0011); *n* = 10/group, results are representative of at least three independent experiments. Column plots display mean with standard of the mean (SEM). Statistical significance for survival data was determined by log-rank Mantel–Cox test while pathological scores were compared by two-tailed Mann–Whitney-test. Asterisks indicate *p* values *p ≤ 0.05, **p ≤ 0.01, and ***p ≤ 0.001, only significant values are shown. Source data are provided as a Source Data file.

Increasing evidence suggests that DCs impact allograft survival age-specifically. It has been shown previously that CD11b^+^CD11c^+^ DCs of young donor origin can extend allograft survival^33^. Moreover, we have shown that depleting old mouse DCs intra-graft prolonged allograft survival^5^. To make these observations, DCs were isolated from young and old C57BL/6 mice (2 × 10^6^, >95% purity) and adoptively transferred into DBA/2J mice i.v. 7 days prior to the engraftment of vascularized heterotopic C57BL/6 cardiac transplants (Fig. 2a). As shown in Fig. 2b, transfer of old mouse CD11b^+^CD11c^+^ DCs significantly reduced median graft survival from 11 days (d) in untreated controls to 8 d (*p* < 0.02). Conversely, adoptive transfer of young mouse CD11b^+^CD11c^+^ DCs into old recipients prolonged graft survival from 9 to 12 d (*p* < 0.01).

Moreover, histological examination of cardiac allografts revealed a dramatic difference between recipients that had received either adoptively transferred DCs from young or old mice. In line with the augmented activation of old mouse CD11b^+^CD11c^+^ DCs in vitro and the accelerated graft rejection, we observed that cardiac allografts of mice that had received adoptively transferred old DCs had multiple inflammatory foci. In contrast, only few inflammatory cells were detected in recipients of young mouse DCs. Accordingly, pathological scores of animals that had received either old or young mouse DCs were significantly different (*p* < 0.05) (Fig. 2c).

Collectively, these data show that DCs are activated and promote CD4^+^ T cell infiltration in an age-specific manner, accelerating allograft rejection.

### DC activation by an age-specific increase in cf-mt-DNA

It is well established that aging is associated with low-grade inflammation, contributing to the development of several diseases, including atherosclerosis, Alzheimer’s disease, and malignancies^34^. Conceptually, inflamm-aging has been linked to continuous stimulation of professional antigen-presenting cells including DCs, a process termed “Garb-aging”, in which clearance of DAMPs is impaired^35^. These processes become even more relevant when responding to injuries such as IRI. Indeed, both aging and IRI are associated with increased DAMP release. In the context of transplantation, cf-mt-DNA has been delineated as a major DAMP, promoting IRI-derived inflammation, however its role in aging remains unknown^36^. Therefore, we investigated whether DAMPs, specifically cf-mt-DNA levels, increase with aging.

**Fig. 3:**
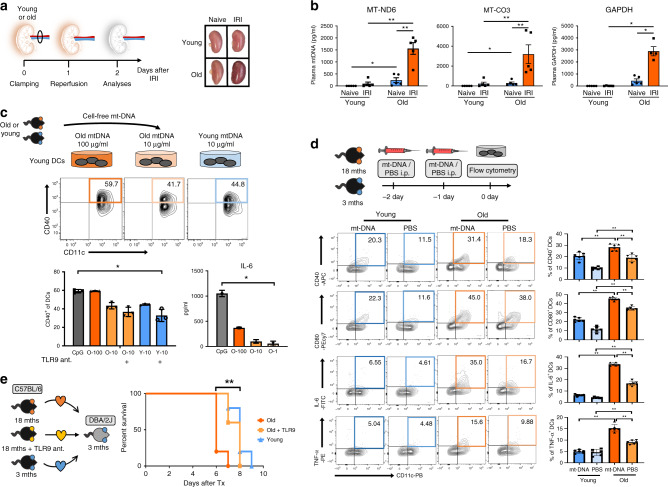
Systemic cf-mt-DNA increases upon IRI in old mice and promotes DC maturation through TLR9. **a** Ischemia reperfusion injury was induced by clamping the renal pedicle of young and old C57Bl/6 (2 and 18 months) mice for 22 min, respectively. IRI and naive animals were euthanized after 48 h and kidneys were procured. The image shows the macroscopic appearance of kidneys directly after IRI. **b** Cell-free mitochondrial DNA (cf-mt-DNA) was quantified in plasma by real-time PCR according to standard curve results (young IRI vs. old IRI for mt-CO3: *p* = 0.0079); Column plots display Mean ± SEM; (*n* = 5 biologically independent animals). **c** Different concentrations of cf-mt-DNA isolated from young and old mice were added to DC cultured from young mice, costimulatory molecule expression was analyzed by flow cytometry with or in absence of a TLR9 antagonist (CpG vs. young + TLR9 ant: *p* = 0.0376), and Il-6 production was measured by ELISA (CpG vs. O1: *p* = 0.0134); statistical significance was determined using Kruskal–Wallis test with Dunn´s post hoc test; (*n* = 3 biologically independent samples). **d** Young or old C57BL/6 mice were injected i.v. with either 30 mg mt-DNA or PBS for 2 consecutive days. Subsequently, costimulatory molecule and cytokine expression of splenic DCs was analyzed by flow cytometry (young + mt-DNA vs. old + mt-DNA: (*p* = 0.0022); (*n* = 6 biologically independent animals). **e** Immediately after receiving a fully mismatched cardiac allograft from 18 months old C57Bl/6 donor mouse, 3 months old DBA2/J recipient mice were treated every 24 h with an i.p. TLR9 antagonist for the follow-up period (*p* = 0.0009), (*n* = 5 biologically independent animals); results are representative of at least three independent experiments. Column plots display mean with standard deviation. Statistical significance was determined using two-tailed Mann–Whitney test. Survival was compared by log-rank Mantel–Cox test. Asterisks indicate *p* values *p ≤ 0.05, **p ≤ 0.01, and ***p ≤ 0.001, only significant values are shown. Source data are provided as a Source Data file.

Cell-free-mt-DNA levels were quantified with primers for the mitochondrially *encoded NADH dehydrogenase subunit 6* (*MT-ND6*) and *cytochrome c oxidase subunit III* (*MT-CO3*) of the electron transport chain^37^. Old mice exhibited increased levels of cf-mt-DNA, while levels were not detectable in young animals (Fig. 3b). These differences became significantly more pronounced after IRI. Notably, old mice that underwent renal IRI (Fig. 3a) had a 15-fold increase in cf-mt-DNA levels compared with young animals (Fig. 3b). Systemic *GAPDH* was absent from serum of old and young naive mice but became detectable in mice subjected to IRI (Fig. 3b).

Cf-mt-DNA has the capacity to induce sterile inflammation as a response to injury through a TLR9 dependent pathway^17^. We therefore tested whether the augmented systemic cf-mt-DNA levels in old mice activate DCs. We did so by culturing DCs isolated from young, naive C57BL/6 mice (3 months) with young and old plasma DNA. Old plasma DNA activated the costimulatory molecules, CD40 and CD80, in an age-specific fashion. Remarkably, after adding ODN 2088, a TLR9 antagonist, the upregulation of costimulatory molecules was attenuated in the presence of old mouse cell-free plasma DNA (Supplementary Fig. 1).

Next, we tested these effects in detail by using isolated mt-DNA at different concentrations and consistently detected a dose-dependent upregulation of CD40. This effect was attenuated when the receptor for mt-DNA was blocked by a TLR9 antagonist (Fig. 3c). Of note, comparable mt-DNA concentrations (10 μg) from either old or young mouse plasma did not impact DC activation, suggesting that cf-mt-DNA quantities, rather than qualitative differences in old animals, determined DC activation (Fig. 3c). We also observed that levels of IL-6, a pro-inflammatory cytokine that is a component of the SASP, underwent a dose-dependent increase in the culture supernatant upon mt-DNA stimulation (Fig. 3c). To delineate the link between mt-DNA accumulation and DC activation in vivo, we administered mt-DNA (i.v.) for 2 consecutive days to young and old C57BL/6 mice and assessed DC activation and pro-inflammatory cytokine production. Notably, CD11c^+^CD11b^+^ DCs from old but not young mice expressed significantly elevated activation markers (CD80, CD40) and pro-inflammatory cytokines (IL-6, TNF-α) subsequent to the transfer of mt-DNA (Fig. 3d). Moreover, in vivo TLR9 blockade in recipients of cardiac allotransplants from old mice prolonged allograft survival to that observed when young mouse hearts had been transplanted (Fig. 3e).

Taken together, old mouse DCs promoted age-specific alloimmune responses, accelerating the rejection of older organs. Moreover, increasing amounts of mt-DNA activated DCs in an age-specific fashion via TLR9.

### cf-mt-DNA from senescent cells accumulates with aging

Mutations of mitochondrial DNA increase with aging^38^. Moreover, dysfunctional mitochondria accumulate^39^, contributing to cellular senescence in vitro and in vivo^40,41^. In addition, accumulation of mitochondrial reactive oxygen species (ROS) has been linked to cellular senescence^42^ with the mitochondrial dysfunction-associated senescence phenotype being of critical importance^43^. Therefore, we next investigated senescent cells as a potential source of increased cell-free mt-DNA levels in old animals and performed IHC stains for the cyclin-dependent kinase inhibitors p21^Cip1^ and p16^Ink4a^, both markers of cellular senescence^9–11^. In addition, we tested murine kidneys for lysosomal-origin-β-galactosidase (SA-β-GAL), an enzyme whose activity is increased in many senescent cells^44^.

**Fig. 4:**
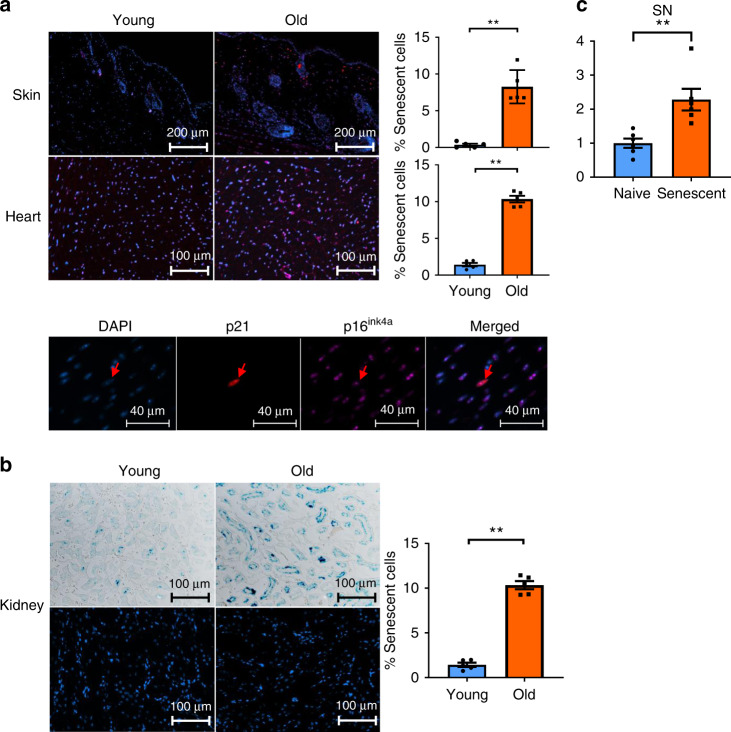
Senescent cells accumulate with aging and are a source of cf-mt-DNA with aging. Skin, hearts, and kidneys were procured from old and young C57BL/6 mice and embedded in paraffin. **a** Skin and hearts were cut into slides and co-stained for p16^Ink4a^, p21^Cip1^, and DAPI; **b** frozen slides of kidneys were made and subsequently stained for sa-β-gal. The percentage of senescent cells was defined as the number of (**a**) p16/p21 double-positive cells or (**b**) sa-β-gal-positive cells of DAPI-stained cells using a confocal microscope (*p* = 0.0079), (*n* = 5 biologically independent animals). **c** Mouse adipocytes were isolated from C57BL/6 mice and senescence induced using 30 serial passages or 10 Gy irradiation. Cf-mt-DNA levels were measured in supernatants by real-time PCR comparing senescent and naive cell cultures (*p* = 0.0022); (*n* = 6 biologcially independent samples); column plots display mean ± SD, results are representative of at least three independent experiments. Statistical significance was determined by using two-tailed Mann–Whitney test. Asterisks indicate *p* values *p ≤ 0.05, **p ≤ 0.01, and ***p ≤ 0.001, ****p ≤ 0.0001, only significant values are shown. Source data are provided as a Source Data file.

Skin and hearts of older mice contained significantly more senescent cells as indicated by p16^Ink4a^/p21^Cip1^ double positivity (Fig. 4a). Similarly, kidneys from older donors had higher numbers of SA-β-Gal-positive cells (Fig. 4b). To link the accumulation of senescent cells to the systemic increase of cf-mt-DNA with aging, we next isolated pre-adipocytes from C57BL/6 mice and irradiated them with 10 Gy to induce senescence. We observed significantly higher yields of mt-DNA in the supernatant of senescent pre-adipocytes (Fig. 4c), suggesting that senescent cells are a key source of the increased cf-mt-DNA with aging.

### Increased cf-mt-DNA in old human organ donors activates DCs

**Fig. 5:**
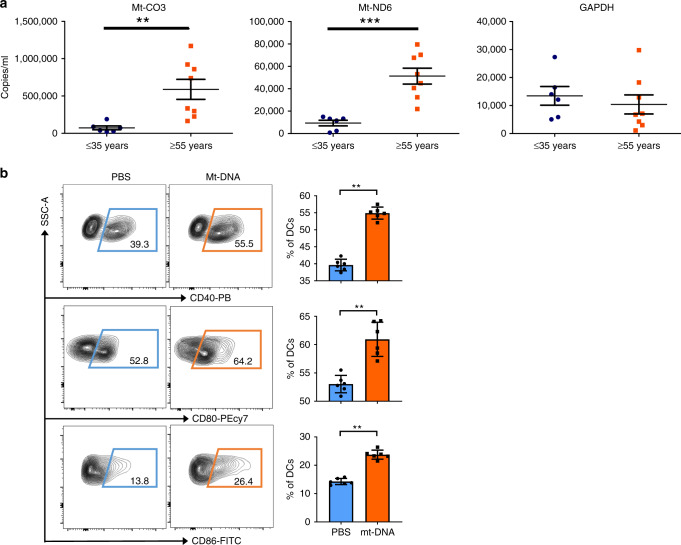
Old human organ donors had increased systemic levels of cf-mt-DNA activating DCs. **a** Cf-mt-DNA was quantified in the plasma of young (<35 years) and old (>55 years) organ donors by RT-PCR using TAQMAN primers for mt-Co3 (*p* = 0.0013) and mt-nd6 (*p* = 0.0007), Scatter plots display mean ± SEM. **b** Dendritic cells were differentiated from isolated PBMC, stimulated with human mt-DNA (10 μg/ml), and costimulatory molecule expression was analyzed using flow cytometry (*p* = 0.0022). Column plots display mean ± SD (*n* = 6 biologically independent samples); results are representative of at least three independent experiments. Statistical significance was determined by using two-tailed Mann–Whitney-test. Asterisks indicate *p* values *p ≤ 0.05, **p ≤ 0.01, and ***p ≤ 0.001, only significant values are shown. Source data are provided as a Source Data file.

Experimentally, we have shown that old mice have augmented systemic levels of cf-mt-DNA, resulting in DC activation and an age-specific pro-inflammatory response. We have also shown that senescent cells are a source of IRI-induced cf-mt-DNA release, causing old mouse DCs to induce Th1 and Th17 T cell-driven alloimmune responses when organs are transplanted from older donors. To test the clinical relevance of these findings, we analyzed cf-mt-DNA in plasma samples of older (>55 years) and younger (<35 years) human organ donors (Clinical characteristics detailed in Supplementary Table 1). Notably, young organ donors had consistently lower plasma levels of cf-mt-DNA compared with old donors (Fig. 5a). Next, we delineated the capacity of human mt-DNA to induce DC activation. When stimulating DCs with human mt-DNA, we observed upregulation of the costimulatory molecules CD40, CD80, and CD86 (Fig. 5b), which are critical for mounting alloimmune responses.

### Senolytics decrease cf-mt-DNA and Th1/Th17 immunogenicity

**Fig. 6:**
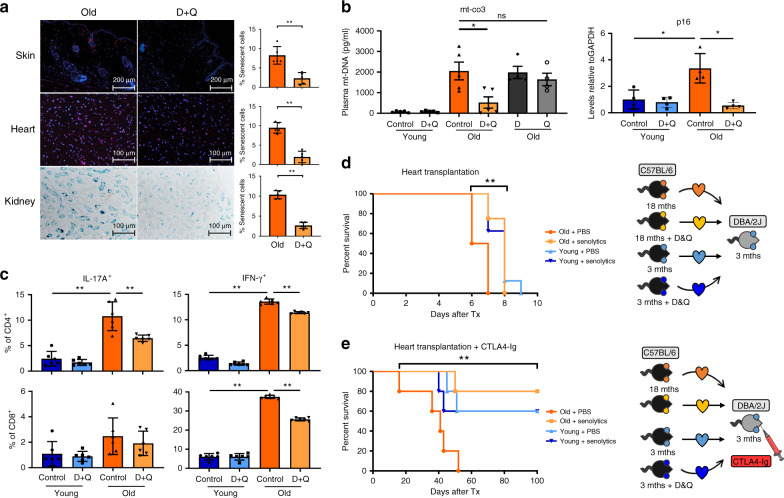
Senolytics decrease the number of senescent cells, reduce cf-mt-DNA levels, alleviate systemic inflammatory immune response after IRI, and prolong cardiac allograft survival. **a** Old C57/B6 mice were treated with senolytics (D and Q, or either D or Q in **b**) on 3 successive days/week. After 1 month, kidney, heart, and skin were procured, stained, and the percentage of senescent cells assessed as described in Fig. 4, (*p* = 0.0079); Column plots display mean ± SD, (*n* = 5 biologically independent animals). **b** Systemic levels of p16^Ink4a^ and cf-mt-DNA were measured by real-time PCR and calculated relative to GAPDH expression (old vs. old + D and Q: *p* = 0.0317); Column plots display mean ± SEM, (*n* = 6 biologically independent animals). **c** Young and old C57BL/6 mice were treated with senolytics for 3 successive days/week for 1 month. Subsequently, IRI was induced in young and old animals; IL-17 and IFN-γ expression of CD4^+^ and CD8^+^ T cells were assessed by flow cytometry (old control vs. old treated: CD4^+^IL-17^+^ (*p* = 0.0087)/CD4^+^IFN-γ^+^ (*p* = 0.0022)/CD8^+^IFN-γ^+^ (*p* = 0.0022); column plots display mean ± SD, (*n* = 6 biologically independent animals). **d** Old and young donor C57BL/6 mice were treated with D and Q or PBS prior to fully mismatched cardiac transplantation and allograft survival was monitored by daily palpation (*p* = 0.0012); (*n* = 8 biologically independent animals). **e** Old and young donor C57BL/6 mice were treated with D and Q or PBS prior to fully mismatched cardiac transplantation while recipient animals were treated with weekly injections of CTLA4-IG following transplantation and allograft survival was monitored by daily palpation (*p* = 0.0064); (*n* = 5 biologically independent animals). Results are representative of at least three independent experiments. Statistical significance was determined by two-tailed Mann–Whitney test. Survival was compared by log-rank Mantel–Cox test. Asterisks indicate *p* values *p ≤ 0.05, **p ≤ 0.01, and ***p ≤ 0.001, ****p ≤ 0.0001, only significant values are shown. Source data are provided as a Source Data file.

To test the consequences of augmented cf-mt-DNA levels with aging in vivo, we next treated animals with senolytics (Dasatinib plus Quercetin), drugs that induce selective apoptosis of senescent cells^11,29^. Treating old C57BL/6 mice (18 months) with Dasatinib (D, 5 mg/kg) plus Quercetin (Q, 50 mg/kg) significantly decreased p16^Ink4a^/p21^Cip1+^ cells in skin and hearts. Moreover, SA-β-Gal-positive cells were significantly decreased in kidneys subsequently to D + Q treatment (Fig. 6a).

Next, we tested effects of senolytics in relevant preclinical disease models. Treatment with D and Q prior to renal IRI reduced systemic levels of cf-mt-DNA in old mice substantially. Moreover, the senescent cell marker p16^*Ink4a*^ was significantly reduced in old mouse kidneys after treatment with senolytics (Fig. 6b). In addition, senolytics reduced systemic levels of pro-inflammatory T cells, including CD8^+^ IFN-γ^+^, CD4^+^IFN-γ^+^, and CD4^+^IL-17^+^ cells in old animals after IRI (Fig. 6c**)**. Notably, administration of either D or Q individually was less effective than the combination of D + Q in reducing cf-mt-DNA levels (Fig. 6b).

### Senolytics prolong old cardiac allograft survival

Next, we examined if clearing senescent cells prolongs graft survival and treated old and young donor mice (*n* = 8) with a single dose of D + Q prior to organ procurement. Senolytics specifically prolonged the survival of cardiac allografts from older donors (Fig. 6d). Differences in graft survival became particularly impressive when applying a clinically relevant immunosuppressive regimen with the co-stimulation blocker CTLA4-Ig, a fusion protein of CLTA-4 and IgG that blocks the interaction of CD80/86 with CD28 on naive T cells. Transplanting organs from old donors that had been treated with D and Q into recipients that were treated with CTLA4-Ig resulted in comparable survival of hearts from old and young donor mice. Moreover, most hearts from treated old donors (80%) survived the observation period (100 days), while organs from old untreated donors stopped working by 37 ± 12 days (Fig. 6e).

These findings emphasize the clinical potential of senolytics for enabling use of organs from older donors for transplantation.

Collectively, our results demonstrate a link between senescent cell clearance and reduced cf-mt-DNA systemic levels in relevant experimental and clinical models. Importantly, clearance of senescent cells in old animals decreased cf-mt-DNA levels, attenuating DC activation, and pro-inflammatory T cell responses. Of clinical relevance, senolytics alleviated the consequences of IRI and significantly prolonged allograft survival.

## Discussion

Our experimental and clinical analyses revealed that the sterile inflammation that occurs with aging is driven, in part, by an immune axis triggered by DAMPs activating DCs that, in turn, stimulate effector T cells. Experimentally, a higher burden of senescent cells was found systemically and in organs from old donors, along with increased cf-mt-DNA in supernatants of these cells. Clinically, levels of cf-mt-DNA were elevated in the peripheral blood of older deceased human organ donors, consistent with studies in healthy elderly individuals^16^. Thus, increased levels of cf-mt-DNA might be reflective of the impaired mitochondrial function in senescent cells.

Cf-mt-DNA has been identified as a driver of age-related cellular senescence. Previously, it has been observed that local administration of senolytics improves pancreatic islet function in immune deficient mice^45^. Here, we conducted extensive mechanistic analysis of alloimmunity and transplant outcomes in experimental models of allogeneic solid organ transplantation. Our findings, supported by data from samples of deceased human organ donors, emphasize the clinical relevance of cellular senescence and cf-mt-DNA for transplant outcomes when using organs from older donors^4^.

Mitochondrial depletion has been shown to abrogate the production of SASP components such as ROS and IL-6^46^. Additional studies have demonstrated the relevance of cf-mt-DNA accumulation and subsequent NLRP3 inflammasome-dependent secretion of IL-1β either by experimental depletion of the autophagic proteins, LC3B and beclin 1, or following depletion of the anti-apoptotic protein, Bcl-2^18,47^. Moreover, pharmacological induction of mitophagy, a subtype of autophagy clearing dysfunctional mitochondrial organelles including mt-DNA, has been successful in improving cardiovascular function with aging and extending murine life span^48^.

We demonstrated that old cell-free plasma DNA and cf-mt-DNA cause DC activation, with augmented expression of IL-6 and the costimulatory molecules, CD40/CD80. We therefore hypothesized that increased amounts of cell-derived cf-mt-DNA drive the maturity of DCs with aging. In adoptive DC transfer experiments, we have previously shown that old DCs can potently induce proliferation and IFN-γ production in allogeneic T cells, resulting in compromised cardiac allograft survival^5^. Findings by others have confirmed that human DNA activates DCs from old humans and simultaneously increases IFNγ levels, driving T cell proliferation^49^. Moreover, exosomal genomic- and mt-DNA derived from human T cells have the capacity to activate DCs upon formation of an immunological synapse^50^. More importantly, using a mouse model of renal IRI that mimics sterile, injury-induced inflammatory responses with aging, we were able to demonstrate a critical role for senescent cells in augmenting systemic immune responses through their SASP. Notably, cf-mt-DNA levels were significantly increased in old animals following renal IRI.

The source of the increased cf-mt-DNA levels could be either due to cellular stress/death or may be due to cell death-independent, selective ROS production, mechanisms that have been proposed previously^47,51,52^. While IFN-γ expression appeared highly elevated in CD8^+^ T cells of old animals, DCs exhibited increased IL-6 levels. These findings are in line with previous reports in DCs from older humans of augmented type I interferon and IL-6 responses to TLR4 ligands^49,53^. Nevertheless, we cannot rule out the possibility that DAMPs, in addition to mt-DNA, may play a direct role in the exacerbated inflammatory responses observed in old animals. Taken together, we demonstrated that senescent cells are a key source of elevated cf-mt-DNA levels with aging. Ischemia reperfusion injury led to a vast increase in mt-DNA with aging, which activated DCs, thereby potentiating effector T cell responses. Of clinical relevance, old deceased organ donors had increased systemic cf-mt-DNA that activated DCs.

In our study, we tested the therapeutic potential of blocking TLR9. In addition, we tested the potential of senolytics, a drug class that acts by transiently disabling the pro-survival pathways that allow senescent cells to evade apoptosis due to their own SASP and pro-apoptotic microenvironment. Senolytics appear to have the potential for slowing age- and cellular senescence-related deterioration of organ function while enhancing healthy life span in old mice^11,29,54–58^. Applying these agents prior to experimental IRI and transplantation reduced the burden of senescent cells in several tissues significantly, while alleviating systemic inflammation and reducing levels of cf-mt-DNA and Th17 and IFNγ^+^ T cells. Most relevantly, we discovered that pretreatment of old donor mice with senolytics prolonged allograft survival, particularly impressively in a clinically relevant model of immunosuppressed recipients. Moreover, a single treatment of recipient mice that received hearts from older donors with TLR9 antagonists prolonged allograft survival.

As with all therapeutic approaches in animal models of human disease, certain aspects may not recapitulate the clinical setting. For example, TLR9 expression in humans is restricted to B cells and plasmacytoid dendritic cells (pDC)^1^, while TLR9 expression in mice is much broader. Moreover, studies in old murine pDC suggested impaired type I IFN pathways during TLR9 activation are due to oxidative stress, albeit with preserved TLR immune responses in conventional murine DCs^59,60^. Further mechanistic studies may evaluate whether senolytics reduce cf-mt-DNA release from organs from older donors not only by clearing senescent cells, but also through enhancing mitophagy.

In a recently published first clinical trial, the senolytics, Dasatinib and Quercetin (D + Q), alleviated physical dysfunction without causing severe side-effects in patients with idiopathic pulmonary fibrosis, a relentlessly progressive fatal and cellular senescence-driven disease^61^. In another study, in patients with systemic sclerosis, Dasatinib reduced levels of skin SASP factors in parallel to reducing relevant gene expression pathways in the skin indicative of the presence of senescent cells^62^. Moreover, we have most recently demonstrated that D + Q decrease senescent cell abundance and associated inflammation in adipose tissue of human patients with diabetic kidney dysfunction and decreased blood SASP factors^63^. Thus, senolytics effectively reduce senescent cell burden and alleviate senescence-induced dysfunction and inflammation, indicating that these agents might eventually prove to be effective in reducing cellular senescence-induced organ failure, inflammation, and dysfunction related to transplanting organs from older donors.

Mechanistically, chronic low-grade inflammation is recognized as a driver of cellular senescence through increased levels of pro-inflammatory cytokines, including IL-6 and TNF-α^64–67^, underscoring the relevance of inflammation as both a result of aging and, simultaneously as a process contributing to the progression of cellular senescence. Indeed, we have shown that the SASP induces cellular senescence both locally and systemically^11^. Cf-mt-DNA, in turn, induced a phenotypic profile reminiscent of senescent cells. Our study revealed that the abundance of cf-mt-DNA may act as both a sensor and effector of tissue damage in an age-dependent fashion. Therefore, we submit that senescent cell-derived cf-mt-DNA drives the immune-stimulatory phenotype of DCs from old mice. This concept is novel and might be of relevance in contributing to the low-grade inflammation that occurs with aging. Our data also strongly suggest a role for senescent cell-derived DAMPs in the exacerbated response to IRI in aging. These mechanisms could be of critical relevance with respect to the observed increased immunogenicity and alloimmunity that occur when organs from older donors are used for transplantation^4^. Previous studies have also noted alterations in DCs with aging and the impact of DCs on multiple conditions, including the pathogenesis of neurodegenerative diseases, auto- and allo-immunity, and infections^68^.

Taken together, our findings support the novel concept that senescent cell accumulation is a key source of cf-mt-DNA, driving alloimmune responses to organs from older donors (Supplementary Fig. 2). Moreover, our data provide a rationale for considering clinical trials treating donors, organs, and/or recipients with senolytic drugs to optimize the use of organs from older donors, helping to close the gap between organ availability and the needs of the many patients currently on transplant waiting lists.

## Methods

### Animals

Young (2 months) C57BL/6 (#027) and DBA/2J (#026) mice were purchased from Charles River Laboratory, Wilmington, MA. Old (18 months) C57BL/6 mice from the same colony were obtained from Charles River Laboratory through the National Institute of Aging (NIA, Bethesda, MD). The study protocol was approved by the Brigham and Women´s Hospital Institutional Animal Care and use Committee (IACUC) (animal protocol #2018N000049). All mice were male, age-matched and experimental and control animals were housed separately. Owing to the exploratory nature of our study, we did not use randomization and blinding. No statistical methods were used to predetermine sample size. All animals were maintained in specific pathogen-free conditions at the Brigham and Women´s Hospital animal facility in accordance with federal, state, and institutional guidelines. Animals were maintained on 12-h light, 12-h dark cycle in facilities with an ambient temperature of 19–22 °C and 40–60% humidity and were allowed free access to water and standard chow. Euthanasia was performed by cervical dislocation following anesthesia with isoflurane (Patterson Veterinary, Devens, MA, USA).

### Deceased organ donors

Approval from the Committee on Human Research at the University of California San Francisco for collecting deceased organ donor blood specimens was not required because deceased donors are not considered human patients under federal law. As governed by the Uniform Anatomical Gift Act, all deceased donors had documentation of separate authorization for donation and research, respectively. Authorization was provided either as first-person authorization (for example, registration with the Department of Motor Vehicles) or legal next-of-kin. Deceased donors were part of a prospective trial, as previously published, and enrolled in the normothermic arm^69^. Samples were convenience samples and collected during donor management, processed onsite, and subsequently stored at −80 degrees for further analysis.

### Adoptive transfer of CD11b^+^ CD11c^+^ subsets

Sorted CD11b^+^ CD11c^+^ DCs from young and old DBA/2J mice were washed extensively in HBSS and injected (2 × 10^6^ in 400–500 µl of HBSS) into DBA/2J mice via the lateral tail vein. After 7 days, mice received vascularized heterotopic C57BL/6 heart transplants, as described below. For ex vivo functional studies, spleens were removed either 7 or 12 days after adoptive transfer of DCs.

### DC isolation and sorting

DCs were isolated from the spleens of naive old and young mice. Spleens were disaggregated and digested for 15 min with 10 ml of type IV collagenase (200 µg/ml; Sigma-Aldrich, St. Louis, MO) in HBSS supplemented with 100 µg/ml DNase (Roche, Mannheim, Germany). After digestion, splenocytes were collected by centrifugation at 500 × *g*, and erythrocytes were lysed by hypotonic shock using 0.15 M NH_4_Cl. DCs were isolated immediately after splenocyte preparation. DCs were enriched from fresh splenocytes by metrizamide (16.5 or 14.5% [w/v], respectively) density centrifugation at 500 × *g* for 15 min at the room temperature (20 °C). For purification by sorting, the buffy coat was labeled with anti-CD11c (#MCD11C28, 1:100), anti-CD11b (#45-0112-82, 1:100), and anti-CD8α (#17-0081-82, 1:100) for 30 min at 4 °C. Cells were washed, incubated for 5 min at 4 °C with cation-free HBSS containing 1% (v/v) FCS and 10 mM EDTA to disaggregate cell clusters, and then resuspended in complete medium. CD11b^+^ CD11c^+^ DC populations with high forward- and side-scatter profiles, were sorted using a Coulter EPICS Elite (Beckman Coulter, Hialeah, FL) to >95% purity. For isolation of CD11c^+^ DCs, single cell suspensions were obtained from hearts of young (8–12 weeks) and old (18 months) C57BL/6 WT mice. Briefly, hearts were procured and washed 3× with Ca_2_^+^- and Mg_2_^+^-free PBS. Tissue was then cut into 5 mm pieces and placed in tissue extraction buffer (5 mM EDTA, 2 mM 2-ME in PBS), and incubated with continuous, brisk stirring at 37 °C for 30 min. The suspension was filtrated through a 70-μm filter. CD11c^+^ DCs were then isolated using EasySep™ Mouse CD11c Positive Selection Kit (Stemcell Technologies, Vancouver, Canada) according to the manufacturer’s protocol.

### Isolation and differentiation of human DCs from PBMCs

Blood was obtained from healthy adult volunteers in accordance with guidelines of and approved by the Institutional Review Board of the Brigham and Women´s Hospital. Informed consent was obtained from each volunteer in accordance with the Declaration of Helsinki. PBMCs were isolated via density gradient centrifugation using SepMate (Stemcell) tubes and lymphoprep (Stemcell) density gradient medium. Briefly, blood was obtained from healthy donors, diluted 1:1 with PBS, and added to the SepMate Tubes containing lymphoprep density gradient medium. Tubes were then centrifuged at 1200 *g* for 10 min and the top layer containing enriched PBMCs was poured off into a new tube. Subsequently, PBMCs were washed twice with PBS. 1–1.5 × 10^6^ PBMC were then plated in a 25 cm^2^ cell culture flask (Corning, New York, NY, USA) in RPMI 1640 (Gibco) supplemented with 10% fetal calf serum, 100 µg/ml streptomycin, 100 U/ml penicillin, 2 mM glutamine, and 1 mM sodium pyruvate for 2.5 h at 37 °C/5% CO_2_. Subsequently, the culture medium containing non-adherent cells and attached cells was washed three times with PBS. Monocytes were then harvested after short centrifugation and 5 × 10^5^ cells/ml re-cultured in 25 ml of culture medium with added 50 ng/ml gm-csf and 30 ng/ml IL-4 (both Promega, Madison, WI, USA) for 6 days. Medium was replaced after 3 days with fresh supplemented medium with re-plating of all non-adherent cells by centrifuging supernatants. DCs were then plated in a 48 well plate at a density of 1 × 10^6^ cells/ml, cultured overnight, and subsequently stimulated with 10 μg/ml isolated mt-DNA/PBS.

### Mixed lymphocyte reaction

Bulk splenocytes or T cells from naive or DC-primed DBA/2J mice were enriched by a single passage through nylon wool columns (45 min at 37 °C) and used as responders. A total of 2 × 10^5^ cells were placed in each well of 96-well round-bottom plates, and varying numbers of gamma-irradiated (20 Gy), sorted CD11b^+^CD11c^+^ DCs from young or old DBA/2J, B6 mice were added as stimulators. In some experiments, human rIL-2 (50 U/ml; Genetics Institute, Cambridge, MA) was added at the start of culture to test for reversal of hypo-responsiveness. Cultures were incubated in complete medium for 72 h unless otherwise specified in a humidified atmosphere of 5% CO_2_ in air.

### Detection of intracellular cytokines

Cytokines were detected intracellularly in responder DBA/2J T cells after 72 h MLR using normal bulk C57BL/6 splenocytes as stimulators (stimulator:responder ratio, 1:1) or CD11b^+^CD11c^+^ DC cells. T cells were then re-stimulated with plate-bound hamster anti-mouse CD3 (BD Pharmingen, #553238, 10 µg/ml) and soluble hamster anti-mouse CD28 (BD Pharmingen, #553295, 10 µg/ml) for 5 h at 37 °C in the presence of Brefeldin A (10 µg/ml; Sigma-Aldrich). Thereafter, cells were washed with 1% (v/v) FCS/PBS, fixed with 4% (w/v) paraformaldehyde (20 min, 4 °C), and permeabilized with 0.15% (w/v) saponin/1% (v/v) FCS/PBS for 15 min at 4 °C. Cells were then labeled and incubated for 30 min at 4 °C with anti-CD3ε (#12-0037-42), anti-CD4 (#11-0042-82), and anti-CD8α (#17-0081-82), (all 1:100). Intracellular cytokines were detected by the addition of conjugated anti-IFN-γ (#48-7311-82), anti-IL-17 (#25-7179-42), anti-IL-6 (#11-7061-41), and anti-TNF-α (#12-7321-82) mAbs (all 1:100) all purchased from ebioscience, San Diego, CA, USA. After staining, cells were washed with 1% (v/v) FCS/PBS, fixed with 1% (w/v) paraformaldehyde, and analyzed immediately using a BD flow cytometer. Cells stained with appropriate isotype-matched Ig (ebioscience) were used as negative controls. Gating strategies are shown in Supplementary Fig. 3.

### ELISA

Splenocytes prepared from DBA/2J mice 5 days after heart transplantation were re-stimulated with bulk donor-type (C57BL/6) splenocytes as described for MLR. Supernatants were harvested after 72 h of co-culture. To assess cytokine production over a discrete period (24 h) at the peak of T cell proliferation, cells were harvested after a 72 h co-culture, washed, and resuspended in fresh complete medium for additional 24-h stimulation with anti-CD3 (10 μg/ml) and anti-CD28 (10 μg/ml) mAbs. ELISA for mouse IFN-γ and IL-17 in culture supernatants was performed using reagents purchased from BD PharMingen following the manufacturer’s recommended procedures.

### Heterotopic heart transplantation

Mice were anesthetized with ketamine (100 mg/kg) and xylazine (10 mg/kg). Using a modified cuff technique, fully vascularized cardiac grafts from young DBA/2 donor mice were heterotopically transplanted into young and old B6 recipients. Hearts were anastomosed to recipient’s common carotid artery and internal jugular vein. Transplantation into the recipient’s cervical region facilitated reliable functional assessment through palpation. Ischemic times were kept consistently at 40 min with an anastomosis time of 12 min. Graft function was measured daily by palpation; allograft rejection was defined as the complete cessation of palpable contractility.

### Immunohistochemistry

Mice were anesthetized with isoflurane, shaved, and the skin over the back was removed with a scalpel. To procure kidneys and hearts, a midline incision was performed, and the rib cage was divided laterally to the thoracic arteries. Kidneys and heart were harvested, respectively, washed with PBS, and embedded in 10% formalin.

For assaying sa-β-gal activity, kidneys were transferred into 30% sucrose after 6 h and incubated overnight at 4 °C. Afterwards, samples were frozen in O.C.T, cut into 5-um sections using a cryostat, and added onto Superfrost Plus Microscope Slides. Activity assayed was performed using a sa-β-gal kit (Cell Signaling, Danvers, MA, USA) according to the manufacturer’s protocol. Slides were covered using Vectashield mounting medium (Vector Laboratories, Burlingame, CA, USA) with DAPI and analyzed using a bright field microscope.

For immunofluorescence, heart and skin were kept in formalin for 18 h and subsequently embedded in paraffin. The paraffin sections were deparaffinized and rehydrated, followed by antigen retrieval using sodium citrate buffer (pH 6). After three washes with TBS, sections were incubated with 5% normal donkey serum (Jackson ImmunoResearch Lab Inc, West Grove PA) for 1 h at the room temperature. Slides were then incubated with mouse anti-p16^Ink4a^ (1:500, Abcam, #ab54210) and rabbit anti-p21^Cip1^ (1:200, Abcam, #ab188224) primary antibodies overnight at 4 °C. Slides were washed three times and incubated with Cy3 conjugated Donkey anti-rabbit secondary antibody (Jackson ImmunoResearch Lab, 1:300, #711-165-152) and Alexa Fluor 647 conjugated Donkey anti-mouse secondary antibody (Invitrogen, 1:300, #A32787). Samples were counterstained with Hoechst dye, then washed three times with TBS, and the slides were mounted with Prolong Gold anti-fade mounting media (Invitrogen).

The percentage of senescent cells was defined as the number of p16/p21 double-positive (Fig. 4a) and sa-β-gal-positive (Fig. 4b)/DAPI-positive cells.

Light and fluorescence microscopy pictures were taken using a Zeiss AxioImager M1 microscope operated through the Axiovision 4.8 and Zen 3.0 blue software and analyzed using ImageJ 1.52.

### Kidney ischemia and reperfusion injury

Young and old B6 mice were subjected to ischemia reperfusion injury by clamping of renal pedicles. Kidneys were exposed through flank incisions and ischemia was induced by bilateral clamping of renal pedicles with nontraumatic microaneurysm clamps (Roboz Surgical Instruments, Gaithersburg, MD) for 22 min. Body temperature was maintained at 36.5–37.3 °C. Successful reperfusion of kidneys was confirmed visually after removal of clamps.

### Flow cytometric analyses

CD3 (#12-0037-42), CD4 (#11-0042-82), CD8α (#17-0081-82), CD11b (#45-0112-82), CD11c (# MCD11C28), CD40 (#17-0401-82), CD80 (#50-112-3308), CD86 (#11-0862-82), MHC-II (#12-5322-81), H2K^b^ (#12-5958-82), and IA^b^β-chain (#12-5320-82) mAbs (all at a dilution of 1:100) were used for immunophenotyping. Draining lymph nodes cells or splenocytes were isolated and suspended in complete RPMI 1640 with 10% FCS at a density of 2.5 × 10^6^/ml. Mononuclear cell suspensions were re-stimulated with PMA (50 ng/ml), ionomycin (500 ng/ml) (Sigma, St. Louis, MO, USA), and treated with Golgi Stop (1 µg/10^6^ cells) (BD PharMingen) for 4–24 h. Cells were procured, washed in staining buffer containing 1% FCS, 0.1% NaN_3_ in PBS, and blocked with anti-CD16/CD32 (#14-0161-82, 1:100) antibodies. Following another wash step, cells were stained with fluorescence-labeled antibodies for 30 min in the dark at 4 °C. Cells were then washed, fixed, and permeabilized using Fix and Perm^®^ cell permeabilization reagents (Caltag Laboratories, Burlingame, CA). Subsequently, cells were stained for intracellular cytokines with conjugated rat anti-mouse IFN-γ (#48-7311-82), 1:100) and IL-17 (#25-7179-42, 1:100) antibodies.

All antibodies were purchased from eBioscience. Flow cytometry measurements were performed using a FACSCalibur system (BD), and data were analyzed using FlowJo (Tree Star, Ashland, OR, USA). Gating strategies are shown in Supplementary Fig. 3.

### Proliferation assays

In vitro proliferation of CD8^+^ and CD4^+^ T cells in MLRs with DCs from young or old mice was determined by CFSE dilution (eBioscience). Briefly, CFSE was dissolved in DMSO to a final stock solution of 10 mmol/l and then resuspended to 10^6^ cells/ml at a concentration of 1 μmol/l. Cells were incubated for 10 min at the room temperature, then for 5 min at 4 °C and subsequently plated. Fluorescence of CFSE stained cells was assessed by flow cytometry with a peak excitation of 494 nm and peak emission of 521 nm.

### Real-time PCR and RNA extraction

DNA extraction from plasma was performed using QIAamp DNA Mini and Blood Mini Kit according to the manufacturer’s protocol (Qiagen, Hilden, Germany). Human samples from young and old organ donors were analyzed in collaboration with CN. DNA extraction from kidney tissue was performed using QIAmp Blood & Tissue (Qiagen, Hilden, Germany). For real-time PCR reactions *human cytochrome c subunit III* (qHsaCEP0055665), *human NADH dehydrogenase 6* (qHsaCEP0055605), *human GAPDH* (qHsaCEP0041396), *mouse cytochrome c subunit III* (qMmuCEP0060078), *mouse NADH dehydrogenase 6* (qMmuCEP0062889), and *mouse GAPDH* (qMmuCEP0039581) measurements were performed using Taqman primers and probes (Bio-Rad Laboratories, Hercules, California, USA). Samples that produced no PCR products after 40 cycles were considered undetectable and the Ct number set to 40 for statistical purposes. Mitochondria were isolated from whole liver tissue of wild-type male C57BL/6 mice using Mitochondria Isolation Kit for Tissue (Thermo Fisher Scientific, Waltham, Massachusetts, USA). Mitochondrial DNA was subsequently extracted from the isolated mitochondria using QIAmp Blood & Tissue (Qiagen, Hilden, Germany). Mt-DNA concentrations were determined by spectrophotometer. A real-time PCR standard curve was created to quantify mt-DNA concentration by using isolated mt-DNA and cytochrome c oxidase subunit III as target. For absolute quantification of mt-DNA levels in human samples, gene-specific synthetic DNA templates (Bio-Rad Laboratories) were used to obtain standard curves. PCR primer sequences can be found in Supplementary Table 2.

For analysis of p16 expression in kidney tissue following IRI, RNA was extracted using RNAqueous extraction kit according to the manufacturer’s protocol (Applied Biosystems, Carlsbad, CA, USA). After successive washes, RNA was eluted and reverse transcription was performed using i-Script^®^ cDNA synthesis kit (Bio-Rad Laboratories, Hercules, CA, USA). For real-time PCR reactions, *p16*^*INK4a*^*/Cdkn2a* (Mm00494449_m1) measurements were performed with Taqman primers and probes from Applied Biosystems. Relative gene expression was determined using the housekeeping gene *GAPDH* (MM99999915_g1) as control.

### Mitochondrial DNA isolation and administration

Mitochondria were isolated from whole liver tissue of wild-type male C57BL/6 mice using Mitochondria Isolation Kit for Tissue (Thermo Fisher Scientific, Waltham, Massachusetts, USA). Mitochondrial DNA was subsequently extracted from the isolated mitochondria using QIAmp Blood & Tissue (Qiagen, Hilden, Germany). Subsequently, young or old C57BL/6 mice were i.v. injected with either 30 mg mt-DNA or PBS for 2 consecutive days prior to flow cytometric analyses.

### Drug administration

Eighteen months old C57BL/6 mice were treated with Dasatinib (D; 5 mg/kg) and Quercetin (Q; 50 mg/kg) (both from Sigma) p.o. for 3 successive days. After 4 days, mice were euthanized, and organs procured for IHC. The same treatment kinetics were applied to animals that underwent IRI to assess systemic cf-mt-DNA levels and inflammation. To monitor cardiac allograft survival, 18 months old C57BL/6 donor mice were treated with a single dose of D (5 mg/kg) and Q (50 mg/kg). Moreover, to model the clinical setting, recipient animals were subjected to weekly i.p. injections with 10 mg/kg CTLA4-Ig (Orencia) in an additional set of experiments. By 24 h, hearts were procured and transplanted. To assess the impact of cf-mt-DNA on alloimmunity, 3 months old DBA2/J recipient mice were treated daily with 30 µg of ODN2088 (Invitrogen, Carlsbad, CA, USA).

### Statistical analysis

Kolmogorov–Smirnov and d’Agostino and Pearson omnibus normality tests were applied to verify Gaussean distribution before using two-sided one-way-ANOVA or Student’s *T* test with Tukey’s post-test or Dunnett’s Multiple Comparison test for proofing statistical significance. Nonparametric data were analyzed using Friedmann test. For in vivo survival data, Kaplan–Meier survival graphs were constructed, and the log-rank comparisons of the groups were used to calculate *p* values. The level of significance was chosen to be at *p* < 0.05 (GraphPadPrism V8, La Jolla, CA, USA).

### Reporting summary

Further information on research design is available in the Nature Research Reporting Summary linked to this article.

## Supplementary information

Supplementary Information

Reporting Summary

## Data Availability

All relevant data supporting the findings of this study are available within the article and its supplementary information or from the authors upon reasonable request. Source data are provided with this paper.

## Source data

Source Data

## References

[1] Korolchuk VI, Miwa S, Carroll B, von Zglinicki T (2017). Mitochondria in cell senescence: is mitophagy the weakest link?. EBioMedicine.

[2] Tullius SG, Rabb H (2018). Improving the supply and quality of deceased-donor organs for transplantation. N. Engl. J. Med.

[3] Klassen DK (2016). The OPTN deceased donor potential study: implications for policy and practice. Am. J. Transpl..

[4] Tullius SG, Milford E (2011). Kidney allocation and the aging immune response. N. Engl. J. Med.

[5] Oberhuber R (2015). CD11c+ dendritic cells accelerate the rejection of older cardiac transplants via interleukin-17A. Circulation.

[6] Tullius SG (2000). Contribution of prolonged ischemia and donor age to chronic renal allograft dysfunction. J. Am. Soc. Nephrol..

[7] Krishnamurthy J (2004). Ink4a/Arf expression is a biomarker of aging. J. Clin. Investig.

[8] Franceschi C (2000). Inflamm-aging. An evolutionary perspective on immunosenescence. Ann. N. Y. Acad. Sci..

[9] Kirkland, J. L. & Tchkonia T. Cellular senescence: a translational perspective. *EBioMedicine* **21**, 21–28 (2017). 10.1016/j.ebiom.2017.04.013 PMC5514381 28416161

[10] Tchkonia T, Zhu Y, van Deursen J, Campisi J, Kirkland JL (2013). Cellular senescence and the senescent secretory phenotype: therapeutic opportunities. J. Clin. Investig.

[11] Xu, M. et al. Senolytics improve physical function and increase lifespan in old age. *Nat. Med*. **24**, 1246–1256 (2018). 10.1038/s41591-018-0092-9 PMC6082705 29988130

[12] Freund A, Orjalo AV, Desprez PY, Campisi J (2010). Inflammatory networks during cellular senescence: causes and consequences. Trends Mol. Med.

[13] Valentijn FA, Falke LL, Nguyen TQ, Goldschmeding R (2018). Cellular senescence in the aging and diseased kidney. J. Cell Commun. Signal.

[14] Baker DJ (2011). Clearance of p16Ink4a-positive senescent cells delays ageing-associated disorders. Nature.

[15] Anderson, R. et al. Length-independent telomere damage drives post-mitotic cardiomyocyte senescence. *Embo J*. **38**, 10.15252/embj.2018100492 (2019). 10.15252/embj.2018100492 PMC6396144 30737259

[16] Pinti M (2014). Circulating mitochondrial DNA increases with age and is a familiar trait: Implications for “inflamm-aging”. Eur. J. Immunol..

[17] Zhang Q (2010). Circulating mitochondrial DAMPs cause inflammatory responses to injury. Nature.

[18] Oka T (2012). Mitochondrial DNA that escapes from autophagy causes inflammation and heart failure. Nature.

[19] Tsuji N (2016). Role of mitochondrial DNA in septic AKI via toll-like receptor 9. J. Am. Soc. Nephrol..

[20] Hu Q, Wood CR, Cimen S, Venkatachalam AB, Alwayn IP (2015). Mitochondrial damage-associated molecular patterns (MTDs) are released during hepatic ischemia reperfusion and induce inflammatory responses. PLoS ONE.

[21] Wang L (2015). Plasma nuclear and mitochondrial DNA levels in acute myocardial infarction patients. Coron. Artery Dis..

[22] Pollara, J., Edwards, R. W., Lin, L., Bendersky, V. A., Brennan, T. V. Circulating mitochondria in deceased organ donors are associated with immune activation and early allograft dysfunction. *JCI Insight*. **3**, 10.1172/jci.insight.121622 (2018). 10.1172/jci.insight.121622 PMC6129133 30089724

[23] Scozzi D (2019). Mitochondrial damage-associated molecular patterns released by lung transplants are associated with primary graft dysfunction. Am. J. Transpl..

[24] Zhang Q, Itagaki K, Hauser CJ (2010). Mitochondrial DNA is released by shock and activates neutrophils via p38 map kinase. Shock.

[25] Xu X (2014). Aging aggravates long-term renal ischemia-reperfusion injury in a rat model. J. Surg. Res..

[26] Fan Q (2013). Aging might augment reactive oxygen species (ROS) formation and affect reactive nitrogen species (RNS) level after myocardial ischemia/reperfusion in both humans and rats. Age.

[27] Lucas DT, Szweda LI (1998). Cardiac reperfusion injury: aging, lipid peroxidation, and mitochondrial dysfunction. Proc. Natl Acad. Sci. USA.

[28] Agrawal A, Agrawal S, Gupta S (2017). Role of dendritic cells in inflammation and loss of tolerance in the elderly. Front Immunol..

[29] Zhu Y (2015). The Achilles’ heel of senescent cells: From transcriptome to senolytic drugs. Aging Cell.

[30] Nowak EC (2009). IL-9 as a mediator of Th17-driven inflammatory disease. J. Exp. Med.

[31] Schmitt V, Rink L, Uciechowski P (2013). The Th17/Treg balance is disturbed during aging. Exp. Gerontol..

[32] Tomihara K (2012). Aging-associated B7-DC+ B cells enhance anti-tumor immunity via Th1 and Th17 induction. Aging Cell.

[33] Min WP (2000). Dendritic cells genetically engineered to express Fas ligand induce donor-specific hyporesponsiveness and prolong allograft survival. J. Immunol..

[34] Sanada F (2018). Source of chronic inflammation in aging. Front. Cardiovasc. Med..

[35] Franceschi C, Garagnani P, Vitale G, Capri M, Salvioli S (2017). Inflammaging and ‘Garb-aging’. Trends Endocrinol. Metab..

[36] Hu Q, Zhou Q, Wu J, Wu X, Ren J (2019). The role of mitochondrial DNA in the development of ischemia reperfusion injury. Shock.

[37] van der Bliek AM, Sedensky MM, Morgan PG (2017). Cell biology of the mitochondrion. Genetics.

[38] Linnane AW, Marzuki S, Ozawa T, Tanaka M (1989). Mitochondrial DNA mutations as an important contributor to ageing and degenerative diseases. Lancet.

[39] Wallace DC, Mitochondrial DNA (2010). mutations in disease and aging. Environ. Mol. Mutagen.

[40] Wang W, Yang X, Lopez de Silanes I, Carling D, Gorospe M (2003). Increased AMP:ATP ratio and AMP-activated protein kinase activity during cellular senescence linked to reduced HuR function. J. Biol. Chem..

[41] Moiseeva O, Bourdeau V, Roux A, Deschenes-Simard X, Ferbeyre G (2009). Mitochondrial dysfunction contributes to oncogene-induced senescence. Mol. Cell Biol..

[42] Passos JF (2010). Feedback between p21 and reactive oxygen production is necessary for cell senescence. Mol. Syst. Biol..

[43] Wiley CD (2016). Mitochondrial dysfunction induces senescence with a distinct secretory phenotype. Cell Metab..

[44] Dimri GP (1995). A biomarker that identifies senescent human cells in culture and in aging skin in vivo. Proc. Natl Acad. Sci. USA.

[45] Pathak S (2018). Polymeric microsphere-facilitated site-specific delivery of quercetin prevents senescence of pancreatic islets in vivo and improves transplantation outcomes in mouse model of diabetes. Acta Biomater..

[46] Correia-Melo C (2016). Mitochondria are required for pro-ageing features of the senescent phenotype. EMBO J..

[47] Shimada K (2012). Oxidized mitochondrial DNA activates the NLRP3 inflammasome during apoptosis. Immunity.

[48] Ren J, Zhang Y (2018). Targeting autophagy in aging and aging-related cardiovascular diseases. Trends Pharm. Sci..

[49] Agrawal A, Tay J, Ton S, Agrawal S, Gupta S (2009). Increased reactivity of dendritic cells from aged subjects to self-antigen, the human DNA. J. Immunol..

[50] Torralba D (2018). Priming of dendritic cells by DNA-containing extracellular vesicles from activated T cells through antigen-driven contacts. Nat. Commun..

[51] Ingelsson B (2018). Lymphocytes eject interferogenic mitochondrial DNA webs in response to CpG and non-CpG oligodeoxynucleotides of class C. Proc. Natl Acad. Sci. USA.

[52] Yousefi S (2008). Catapult-like release of mitochondrial DNA by eosinophils contributes to antibacterial defense. Nat. Med.

[53] Agrawal A (2007). Altered innate immune functioning of dendritic cells in elderly humans: a role of phosphoinositide 3-kinase-signaling pathway. J. Immunol..

[54] Roos, C. M. et al. Chronic senolytic treatment alleviates established vasomotor dysfunction in aged or atherosclerotic mice. *Aging Cell*, 10.1111/acel.12458 (2016). 10.1111/acel.12458 PMC5013022 26864908

[55] Palmer, A. K. et al. Targeting senescent cells alleviates obesity-induced metabolic dysfunction. *Aging Cell*. **18**, e12950 (2019). 10.1111/acel.12950 PMC6516193 30907060

[56] Moncsek, A. et al. Targeting senescent cholangiocytes and activated fibroblasts with Bcl-xL inhibitors ameliorates fibrosis in Mdr2-/- mice. *Hepatology* **67**, 247–259 (2017). 10.1002/hep.29464 PMC5739965 28802066

[57] Musi N (2018). Tau protein aggregation is associated with cellular senescence in the brain. Aging Cell.

[58] Ogrodnik M (2017). Cellular senescence drives age-dependent hepatic steatosis. Nat. Commun..

[59] Tesar BM (2006). Murine [corrected] myeloid dendritic cell-dependent toll-like receptor immunity is preserved with aging. Aging Cell.

[60] Stout-Delgado HW, Yang X, Walker WE, Tesar BM, Goldstein DR (2008). Aging impairs IFN regulatory factor 7 up-regulation in plasmacytoid dendritic cells during TLR9 activation. J. Immunol..

[61] Justice JN (2019). Senolytics in idiopathic pulmonary fibrosis: results from a first-in-human, open-label, pilot study. EBioMedicine.

[62] Martyanov, V., Whitfield, M. L. & Varga, J. Senescence signature in skin biopsies from systemic sclerosis patients treated with senolytic therapy: potential predictor of clinical response? *Arthritis Rheumatol*. **71**, 1766–1767 (2019). 10.1002/art.40934 PMC7863581 31112009

[63] Hickson LJ (2019). Senolytics decrease senescent cells in humans: preliminary report from a clinical trial of Dasatinib plus Quercetin in individuals with diabetic kidney disease. EBioMedicine.

[64] Kandhaya-Pillai R (2017). TNFα-senescence initiates a STAT-dependent positive feedback loop, leading to a sustained interferon signature, DNA damage, and cytokine secretion. Aging.

[65] Braumuller H (2013). T-helper-1-cell cytokines drive cancer into senescence. Nature.

[66] Kim KS, Kang KW, Seu YB, Baek SH, Kim JR (2009). Interferon-gamma induces cellular senescence through p53-dependent DNA damage signaling in human endothelial cells. Mech. Ageing Dev..

[67] Jurk D (2014). Chronic inflammation induces telomere dysfunction and accelerates ageing in mice. Nat. Commun..

[68] Ciaramella A (2016). Myeloid dendritic cells are decreased in peripheral blood of Alzheimer’s disease patients in association with disease progression and severity of depressive symptoms. J. Neuroinflammation.

[69] Niemann CU, Malinoski D (2015). Therapeutic hypothermia in deceased organ donors and kidney-graft function. N. Engl. J. Med.

