# Supplementary material for: Senolytics prevent mt-DNA-induced inflammation and promote the survival of aged organs following transplantation
Source: Nat Commun. 2020 Aug 27;11:4289. doi: 10.1038/s41467-020-18039-x (PMC7453018; doi:10.1038/s41467-020-18039-x)
Supplement: Supplementary file 1 — Supplementary Information [file 41467_2020_18039_MOESM1_ESM.pdf]

Supplementary Material

Senolytics Prevent mt-DNA-Induced Inflammation and Promote the Survival of Aged Organs Following Transplantation

Iske et. al

Supplementary Figures

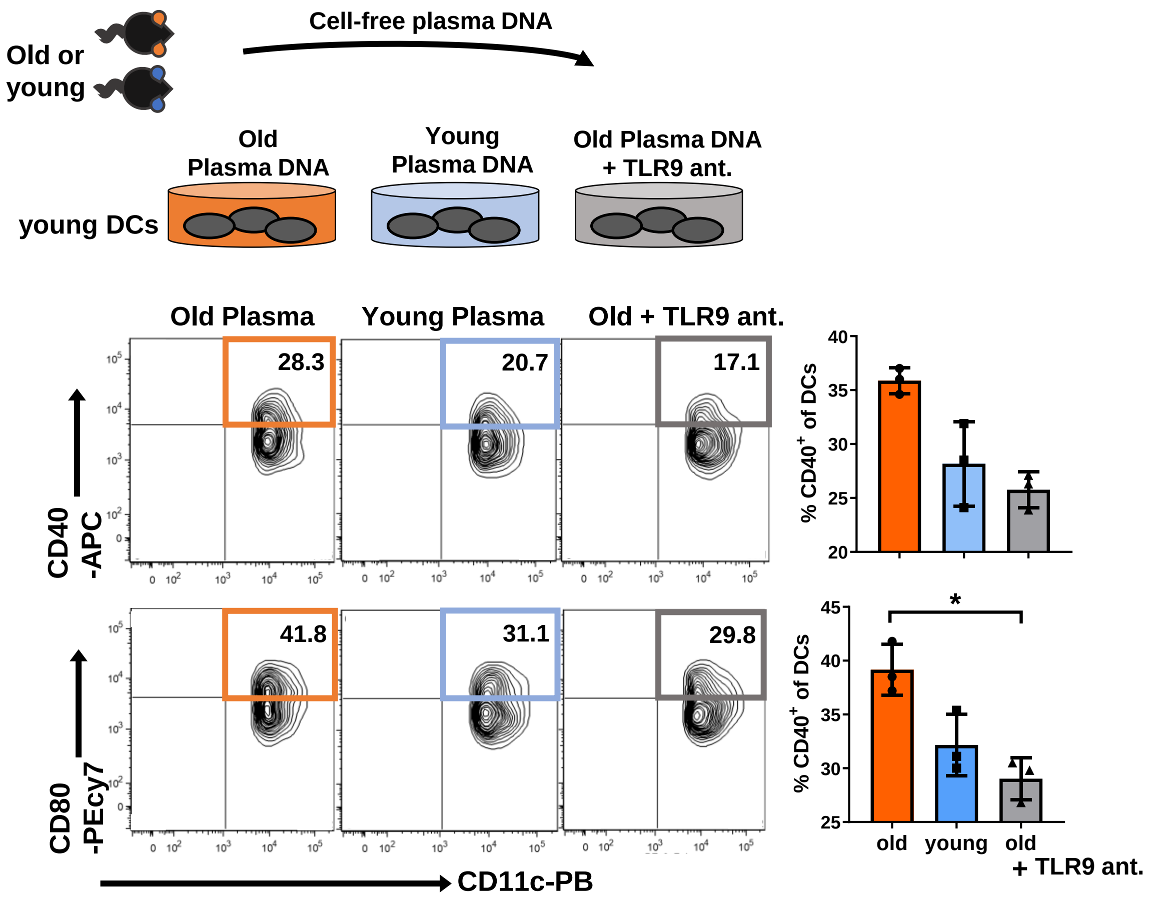

Supplementary Figure 1. Plasma-derived DNA from old mice promotes costimulatory molecule expression on DCs via TLR9

Plasma DNA from young or old mice was added to cultures of DCs from young mice and costimulatory cytokine expression was assessed by flow cytometry with or in absence of a TLR9 antagonist (old vs. old+TLR9 ant: CD40, p=0.0512 /CD80, p=0.0338), n=3 biologically independent samples, Column plots display mean ±SD, experiments are representative of three independent experiment. Statistical significance was determined using one-way Kruskal-Wallis test with Dunn´s post hoc test. Asterisks indicate p-values * = p≤0.05, only significant values are shown.

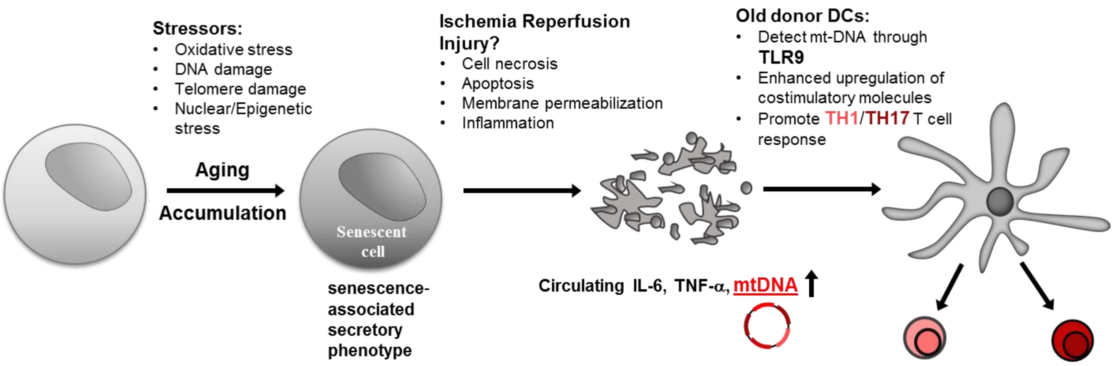

Supplementary Figure 2.
Senescent cell build-up and cf-mt-DNA accumulation at the crossroad of immunogenicity, inflammation, and alloimmunity.

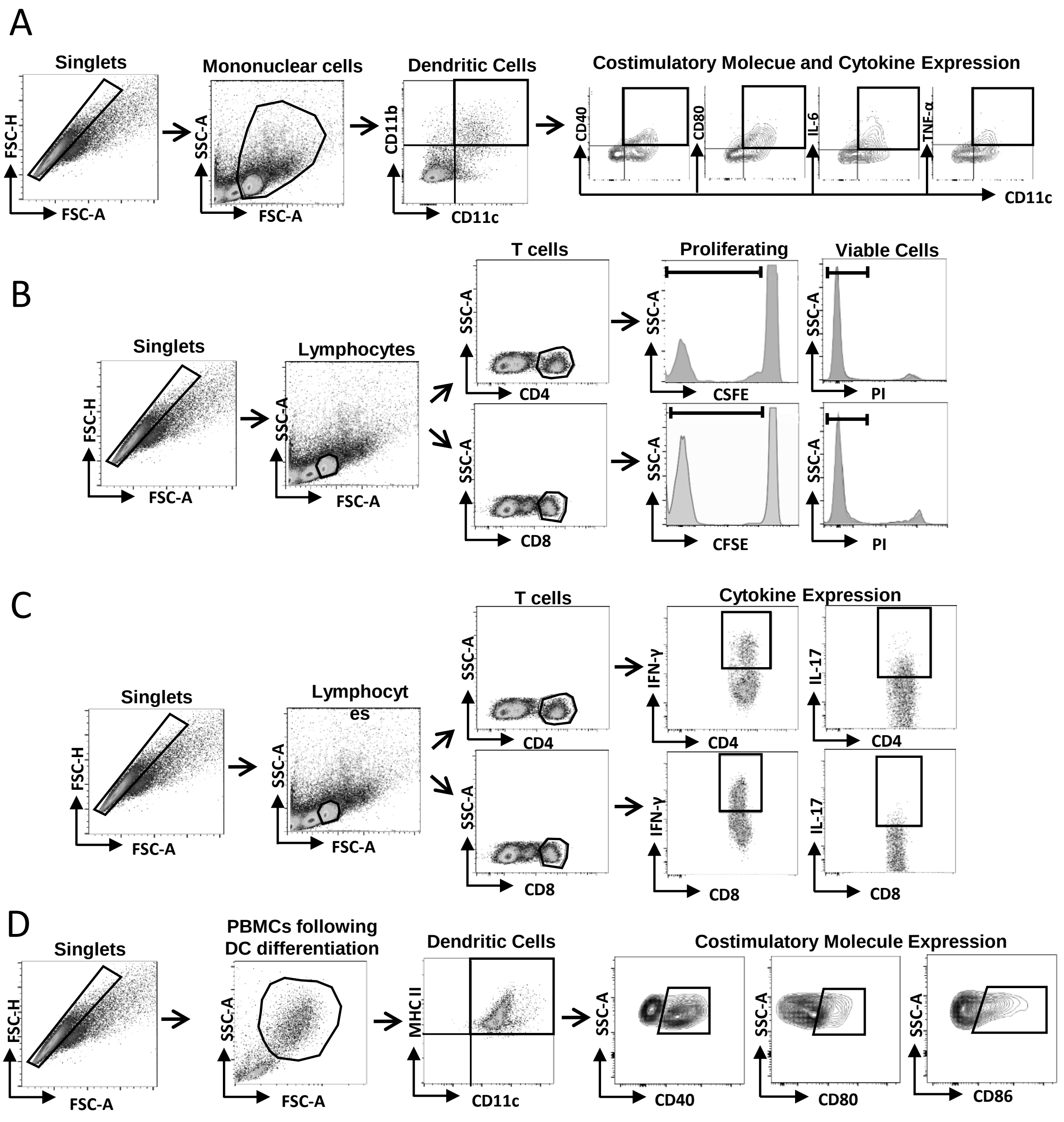

Supplementary Figure 3. Gating Strategies used to analyze flow cytometric data
(A) Gating strategy to analyze DC frequencies in lymph nodes and spleens (Fig. 1A) and costimulatory molecule and cytokine expression (Fig.1B, Fig. 3C and Fig. 3D).
(B) Gating strategy to analyze T cell proliferation and viability (Fig.1C).
(C) Gating strategy to analyze T cell derived cytokine expression (Fig.1D and Fig.6C). **(D)** Gating strategy to analyze costimulatory molecule expression on human dendritic cells differentiated from PBMCs (Fig. 5B).

	Demographic characteristics of organ donors	
	Young (< 33 years old)	Old (>55 years old)
Age distribution	19 – 35	55 – 66
Median age	27.5	61
Sex	♀: 3 (50%)<br>♂: 3 (50%)	♀: 6 (75%)<br>♂: 2 (25%)
Cause of death	4x head trauma<br>1x CVA<br>1x unknown	7x CVA<br>1x Anoxia

Supplementary Table 1.
Characteristics of deceased organ donors.

Primer List

Name	Unique Assay ID	Chromosome location	Unique Gene ID	Amplification Context Sequence
human mitochondrially encoded cytochrome c oxidase III	qHsaCEP0055665	MT:9737-9882	not available	AGCCTCAGAGTACTTCGAGTCTCCCTTCACCATTTCCGACGGCATCTACGGCTCAACATTTTTTGTAGCCACAGGCTTCCACGGACTTCACGTCATTATTGGCTCAACTTTCCTCACTATCTGCTTCATCCGCCAACTAATATTTC
human mitochondrially encoded NADH dehydrogenase 6	qHsaCEP0055605	MT:14250-14382	not available	CACCAATAGGATCCTCCCGAATCAACCCTGACCCCTCTCCTTCATAAATTATTCAGCTTCCTACACTATTAAAGTTTACCACAACCACCACCCCATCATACTCTTTCACCCACAGCACCAATCCTACCTCCAT
human glyceraldehyde-3-phosphate dehydrogenase	qHsaCEP0041396	12:6647267-6647413	Hs.544577	GTATGACAACGAATTTGGCTACAGCAACAGGGTGGTGGACCTCATGGCCCACATGGCCTCCAAGGAGTAAGACCCCTGGACCACCAGCCCCAGCAAGAGCACAAGAGGAAGAGAGAGACCCTCACTGCTGGGGAGTCCCTGCCACAC
mouse mitochondrially encoded cytochrome c oxidase III	qMmuCEP0060078	MT:9135-9279	not availalbe	CAAGCTTCAGAATACTTTGAAACATCATTCTCCATTTCAGATGGTATCTATGGTTCTACATTCTTCATGGCTACTGGATTCCATGGACTCCATGTAATTATTGGATCAACATTCCTTATTGTTTGCCTACTACGACAACTAAAAT
mouse mitochondrially encoded NADH dehydrogenase 6	qMmuCEP0062889	MT:13632-13746	not availalbe	AACTATATATTGCCGCTACCCCAATCCCTCCTTCCAACATAACTCCAACATCATCAACCTCATACATCAACCAATCTCCCAAACCATCAAGATTAATTACTCCAACTTCATC ATA
mouse glyceraldehyde-3-phosphate dehydrogenase	qMmuCEP0039581	6:125162278-125162382	Mm.304088	TGGGAGTTGCTGTTGAAGTCGCAGGAGACAACCTGGTCCTCAGTGTAGCCCAAGATGCCCTTCAGTGGGCCCTCAGATGCCTGCTTCACCACCTTCTTGATGTCA
mouse p16INK4a/Cdkn2a	Mm00494449_m1	Chr.4: 89274473 - 89294619 on Build GRCm38	Mm.4733	CATAGCTTCAGCTCAAGCACGCCCAGGGCCCTGGAACTTCGCGGCCAATCCCAAG
mouse GAPDh	MM99999915_g1	Chr.6: 125161338 - 125166511 on Build GRCm38	Mm.304088	GGAGAGTGTTTCCTCGTCCCGTAGACAAAATGGTGAAGGTCGGTGTGAACGGATTTGGCCGTATTGGGCGCCTGGTCACCAGGGCTGCCATTTGCAGTGGCAAAGTG

Supplementary Table 2. List of Primers used in this study
