# Supplementary material for: Senolytics prevent mt-DNA-induced inflammation and promote the survival of aged organs following transplantation
Source: Nat Commun. 2020 Aug 27;11:4289. doi: 10.1038/s41467-020-18039-x (PMC7453018; doi:10.1038/s41467-020-18039-x)
Supplement: Supplementary file 2 — Reporting Summary [file 41467_2020_18039_MOESM2_ESM.pdf]

nature research

Corresponding author(s): Stefan G. Tullius

Last updated by author(s): Jul 24, 2020

Reporting Summary

Nature Research wishes to improve the reproducibility of the work that we publish. This form provides structure for consistency and transparency in reporting. For further information on Nature Research policies, see our Editorial Policies and the Editorial Policy Checklist.

Statistics

For all statistical analyses, confirm that the following items are present in the figure legend, table legend, main text, or Methods section.

n/a	Confirmed	
☐	☒	The exact sample size (n) for each experimental group/condition, given as a discrete number and unit of measurement
☐	☒	A statement on whether measurements were taken from distinct samples or whether the same sample was measured repeatedly
☐	☒	The statistical test(s) used AND whether they are one- or two-sided<br>*Only common tests should be described solely by name; describe more complex techniques in the Methods section.*
☒	☐	A description of all covariates tested
☒	☐	A description of any assumptions or corrections, such as tests of normality and adjustment for multiple comparisons
☐	☒	A full description of the statistical parameters including central tendency (e.g. means) or other basic estimates (e.g. regression coefficient) AND variation (e.g. standard deviation) or associated estimates of uncertainty (e.g. confidence intervals)
☐	☒	For null hypothesis testing, the test statistic (e.g. F, t, r) with confidence intervals, effect sizes, degrees of freedom and P value noted<br>*Give P values as exact values whenever suitable.*
☒	☐	For Bayesian analysis, information on the choice of priors and Markov chain Monte Carlo settings
☒	☐	For hierarchical and complex designs, identification of the appropriate level for tests and full reporting of outcomes
☒	☐	Estimates of effect sizes (e.g. Cohen's d, Pearson's r), indicating how they were calculated

Our web collection on statistics for biologists contains articles on many of the points above.

Software and code

Policy information about availability of computer code

Data collection	No software was used for data collection.
Data analysis	FlowJo V9 software (TreeStar) Graph Pad Prism V8 (GraphPad Software)<br>software, ZEN 3.0 blue (Zeiss), Axiovision 4.8 (Zeiss), ImageJ 1.52 (National Institute of Health).

For manuscripts utilizing custom algorithms or software that are central to the research but not yet described in published literature, software must be made available to editors and reviewers. We strongly encourage code deposition in a community repository (e.g. GitHub). See the Nature Research guidelines for submitting code & software for further information.

Data

Policy information about availability of data

All manuscripts must include a data availability statement. This statement should provide the following information, where applicable:
- Accession codes, unique identifiers, or web links for publicly available datasets
- A list of figures that have associated raw data
- A description of any restrictions on data availability

All relevant data supporting the findings of this study are available within the article and its supplementary information provided as a source data file or from the authors upon reasonable request.

Field-specific reporting

Please select the one below that is the best fit for your research. If you are not sure, read the appropriate sections before making your selection.

- [x] Life sciences
- [ ] Behavioural & social sciences
- [ ] Ecological, evolutionary & environmental sciences

For a reference copy of the document with all sections, see nature.com/documents/nr-reporting-summary-flat.pdf

Life sciences study design

All studies must disclose on these points even when the disclosure is negative.

Sample size	For in vitro studies, sample size was determined based on preliminary data, variability of the results and previous literature, usually with between 3 and 7 technical replicates per experiment (Krenzien et al, AJT 2017 / Oberhuber et al, Circulation 2015). For in vivo studies, sample size was calculated using G*Power 3.1. (Heinrich Heine Universität Düsseldorf, Düsseldorf, Germany)
Data exclusions	No data were excluded.
Replication	All results shown are representative of at least three independent experiments with successfull replication.
Randomization	Owing to the exploratory nature of our study, we did not use randomization and blinding.
Blinding	Owing to the exploratory nature of our study, we did not use randomization and blinding.

Reporting for specific materials, systems and methods

We require information from authors about some types of materials, experimental systems and methods used in many studies. Here, indicate whether each material, system or method listed is relevant to your study. If you are not sure if a list item applies to your research, read the appropriate section before selecting a response.

Materials & experimental systems

n/a	Involved in the study	
[ ]	[x]	Antibodies
[x]	[ ]	Eukaryotic cell lines
[x]	[ ]	Palaeontology and archaeology
[ ]	[x]	Animals and other organisms
[ ]	[x]	Human research participants
[x]	[ ]	Clinical data
[x]	[ ]	Dual use research of concern

Methods

n/a	Involved in the study	
[x]	[ ]	ChIP-seq
[ ]	[x]	Flow cytometry
[x]	[ ]	MRI-based neuroimaging

Antibodies

Antibodies used

CD3, CD4, CD8α, CD11b, CD11c, CD40, CD80, CD86, MHC-II, IFN-y, IL-17, p21, p16ink4a, Cy3 conjugated Donkey anti-rabbit secondary antibody, Alexa Fluor 647 conjugated Donkey anti-mouse secondary antibody, anti-CD16/CD32, H2KbIAb -chain mAb were used in this study.

Validation

All antibodies used for flow cytometry are from ebioscience:
Anti-Mouse:
CD3 - PE (OKT) - Catalog # 12-0037-42 - https://www.thermofisher.com/antibody/product/CD3-Antibody-clone-OKT3-Monoclonal/12-0037-42
CD4 - FITC (RM4-5) - Catalog # 11-0042-82 - https://www.thermofisher.com/antibody/product/CD4-Antibody-clone-RM4-5-Monoclonal/11-0042-82
CD8a - APC (53-6.7) - Catalog # 17-0081-82 - https://www.thermofisher.com/antibody/product/CD8a-Antibody-clone-53-6-7-Monoclonal/17-0081-82
IFN-y - Pacific Blue (XMG1.2) - Catalog # 48-7311-82 - https://www.thermofisher.com/antibody/product/IFN-gamma-Antibody-clone-XMG1-2-Monoclonal/48-7311-82
IL-17 - PE-cy7 (eBio64DEC17) - Catalog # 25-7179-42 - https://www.thermofisher.com/antibody/product/IL-17A-Antibody-clone-eBio64DEC17-Monoclonal/25-7179-42
Il-6 - FITC (MP5-20F3) - Catalog # 11-7061-41 - https://www.thermofisher.com/antibody/product/IL-6-Antibody-clone-MP5-20F3-Monoclonal/11-7061-41
TNF-alpha - PE (MP6-XT22) - Catalog # 12-7321-82 - https://www.thermofisher.com/antibody/product/TNF-alpha-Antibody-clone-MP6-XT22-Monoclonal/12-7321-82
CD11b - PerCP (M1/70) - Catalog # 45-0112-82 - https://www.thermofisher.com/antibody/product/CD11b-Antibody-clone-M1-70-Monoclonal/45-0112-82

CD11c - Pacific Blue (N418) - Catalog # MCD11C28 - https://www.thermofisher.com/antibody/product/CD11c-Antibody-clone-N418-Monoclonal/MCD11c28
MHC II - PE (NIMR-4) - Catalog # 12-5322-81 - https://www.thermofisher.com/antibody/product/MHC-Class-II-I-A-Antibody-clone-NIMR-4-Monoclonal/12-5322-81
CD40 - APC (1C10) - Catalog # 17-0401-82 - https://www.thermofisher.com/antibody/product/CD40-Antibody-clone-1C10-Monoclonal/17-0401-82
CD80 - PE-cy7 (16-10A1) - Catalog # 50-112-3308 - https://www.fishersci.com/shop/products/cd80-b7-1-armenian-hamster-anti-mouse-pe-cy7-clone-16-10a1-ebioscience-1/501123308
CD86 - FITC (GL1) - Catalog # 11-0862-82 - https://www.thermofisher.com/antibody/product/CD86-B7-2-Antibody-clone-GL1-Monoclonal/11-0862-82
H2Kb – PE, AF6-88.5.5.3 - Catalog # 12-5958-82 - https://www.thermofisher.com/antibody/product/MHC-Class-I-H-2Kb-Antibody-clone-AF6-88-5-5-3-Monoclonal/12-5958-82
IAb –chain-PE (AF6-120.1) - Catalog # 12-5320-82 - https://www.thermofisher.com/antibody/product/MHC-Class-II-I-Ab-Antibody-clone-AF6-120-1-Monoclonal/12-5320-82
CD16/CD32 (93) - Catalog # 14-0161-82 - https://www.thermofisher.com/antibody/product/CD16-CD32-Antibody-clone-93-Monoclonal/14-0161-82
Human:
CD11b – APC (M1/70) - Catalog # 17-0112-82 - https://www.thermofisher.com/antibody/product/CD11b-Antibody-clone-M1-70-Monoclonal/17-0112-82
CD11c – PerCP (N418) - Catalog # 45-0114-82 - https://www.thermofisher.com/antibody/product/CD11c-Antibody-clone-N418-Monoclonal/45-0114-82
CD40 - PB (5C3) - Catalog # 48-0409-42 - https://www.thermofisher.com/antibody/product/CD40-Antibody-clone-5C3-Monoclonal/48-0409-42
CD80 - PE-Cy7 (2D10.4) - Catalog # 15-0809-42 - https://www.thermofisher.com/antibody/product/CD80-B7-1-Antibody-clone-2D10-4-Monoclonal/15-0809-42
CD86 - FITC (BU36) - Catalog # MHCD8601 - https://www.thermofisher.com/antibody/product/CD86-Antibody-clone-BU63-Monoclonal/MHCD8601

antibodies used for microscopy:
p21 (rabbit) - Abcam, Catalog # ab188224 - https://www.abcam.com/p21-antibody-epr18021-ab188224.html
p16ink4a (mouse) - Abcam, Catalog # ab54210 - https://www.abcam.com/cdkn2ap16ink4a-antibody-2d9a12-ab54210.html
Cy3 conjugated Donkey anti-rabbit secondary antibody – Catalog # 711-165-152 - Jackson ImmunoResearch - https://www.jacksonimmuno.com/catalog/products/711-165-152
Alexa Fluor 647 conjugated Donkey anti-mouse secondary antibody - Catalog # A32787- https://www.thermofisher.com/antibody/product/Donkey-anti-Mouse-IgG-H-L-Highly-Cross-Adsorbed-Secondary-Antibody-Polyclonal/A32787

antibodies for cell culture:
anti-CD3 (hamster, 500A2) - BD Pharmingen, Catalog # 553238 - https://www.bdbiosciences.com/eu/applications/research/t-cell-immunology/th-1-cells/surface-markers/mouse/purified-hamster-anti-mouse-cd3e-500a2/p/553238
anti-CD28 (hamster, 37.51) - BD Pharmingen, Catalog # 553295 - https://www.bdbiosciences.com/eu/applications/research/t-cell-immunology/regulatory-t-cells/surface-markers/mouse/purified-hamster-anti-mouse-cd28-3751/p/553295

Animals and other organisms

Policy information about studies involving animals; ARRIVE guidelines recommended for reporting animal research

Laboratory animals	Young (2 mths) C57BL/6 (B6) and DBA/2J (all male) mice were purchased from Charles River Laboratory, Wilmington, MA. Old (18 mths) C57BL/6 mice (also male) from the same colony were obtained from Charles River Laboratory through the National Institute of Aging (NIA, Bethesda, MD)
Wild animals	No wild animals were used in this study.
Field-collected samples	No field-collected samples were used in this study.
Ethics oversight	The study protocol was approved by the Brigham and Women´s Hospital Institutional Animal Care and use Committee (IACUC) (animal protocol #2018N000049)

Note that full information on the approval of the study protocol must also be provided in the manuscript.

Human research participants

Policy information about studies involving human research participants

Population characteristics	Deceased donors were part of a prospective trial, as previously published, and enrolled in the normothermic arm (Niemann et al, N Eng J Med 2015). Further information regarding patient characteristics are provided in Supplementary Table 1. PBMC Donor were healthy adult volunteers.
Recruitment	Deceased donors were part of a prospective trial, as previously published, and enrolled in the normothermic arm (Niemann et al, N Eng J Med 2015). PBMC Donor were healthy adult volunteers who gave informed consent to blood collection.

Ethics oversight	Approval from the Committee on Human Research at the University of California San Francisco for collecting deceased organ donor blood specimens was not required because deceased donors are not considered human subjects patients under federal law. As governed by the Uniform Anatomical Gift Act (UAGA), all deceased donors had documentation of separate authorization for donation and research, respectively. Authorization was provided either as first-person authorization (for example, registration with the Department of Motor Vehicles, DMV) or legal next-of-kin.<br>Blood was obtained from healthy adult volunteers in accordance with guidelines of and approved by the Institutional Review Board of the Brigham and Women´s Hospital. Informed consent was obtained from each volunteer in accordance with the Declaration of Helsinki.

Note that full information on the approval of the study protocol must also be provided in the manuscript.

Flow Cytometry

Plots

Confirm that:

- [x] The axis labels state the marker and fluorochrome used (e.g. CD4-FITC).
- [x] The axis scales are clearly visible. Include numbers along axes only for bottom left plot of group (a 'group' is an analysis of identical markers).
- [x] All plots are contour plots with outliers or pseudocolor plots.
- [x] A numerical value for number of cells or percentage (with statistics) is provided.

Methodology

Sample preparation	Cells were procured, washed in staining buffer containing 1% FCS, 0.1% NaN3 in PBS. Following another wash step, cells were stained with fluorescence-labeled antibodies for 30 mins in the dark at 4°C. Cells were then washed, fixed, and permeabilized using Fix and Perm® cell permeabilization reagents (Caltag Laboratories, Burlingame, CA). Subsequently, cells were stained for intracellular cytokines.
Instrument	FACSCalibur system
Software	FlowJo (Tree Star, Ashland, OR, USA)
Cell population abundance	Spleens were disaggregated and digested for 15 min with 10 ml of type IV collagenase (200 µg/ml; Sigma-Aldrich, St. Louis, MO) in HBSS supplemented with 100 µg/ml DNase (Roche, Mannheim, Germany). After digestion, splenocytes were collected by centrifugation at 500 ×g, and erythrocytes were lysed by hypotonic shock using 0.15 M NH4Cl. DCs were isolated immediately after splenocyte preparation. DCs were enriched from fresh splenocytes by metrizamide (16.5 or 14.5% (w/v), respectively) density centrifugation at 500 ×g for 15 min at room temperature (20°C). For purification by sorting, the buffy coat was labeled with anti-CD11c, anti-CD11b, and anti-CD8α for 30 min at 4°C. Cells were washed, incubated for 5 min at 4° C with cation-free HBSS containing 1% (v/v) FCS and 10 mM EDTA to disaggregate cell clusters, and then resuspended in complete medium. CD11b+ CD11c+ DC populations with high forward- and side-scatter profiles, were sorted using a Coulter EPICS Elite (Beckman Coulter, Hialeah, FL) to >95% purity.
Gating strategy	To set the gates, flow cytometry dot plots were based on comparison with isotype controls, fluorescence minus one, permeabilized and unpermeabilized unstained cells.

- [x] Tick this box to confirm that a figure exemplifying the gating strategy is provided in the Supplementary Information.
